# Genetic and physical interactions between Polη and Rev1 in response to UV-induced DNA damage in mammalian cells

**DOI:** 10.1038/s41598-021-00878-3

**Published:** 2021-11-01

**Authors:** Tonghui Bi, Xiaohong Niu, Chunping Qin, Wei Xiao

**Affiliations:** 1 Beijing Key Laboratory of DNA Damage Responses and College of Life Sciences, Capital Normal University, Beijing, 100048 China; 2 Department of Biochemistry, Microbiology and Immunology, University of Saskatchewan, Saskatoon, SK S7N 5E5 Canada

**Keywords:** Genomic instability, Stress signalling

## Abstract

In response to UV irradiation, translesion DNA synthesis (TLS) utilizes specialized DNA polymerases to bypass replication-blocking lesions. In a well-established polymerase switch model, Polη is thought to be a preferred TLS polymerase to insert correct nucleotides across from the thymine dimer, and Rev1 plays a scaffold role through physical interaction with Polη and the Rev7 subunit of Polζ for continual DNA synthesis. Defective Polη causes a variant form of xeroderma pigmentosum (XPV), a disease with predisposition to sunlight-induced skin cancer. Previous studies revealed that expression of Rev1 alone is sufficient to confer enhanced UV damage tolerance in mammalian cells, which depends on its physical interaction with Polζ but is independent of Polη, a conclusion that appears to contradict current literature on the critical roles of Polη in TLS. To test a hypothesis that the Rev1 catalytic activity is required to backup Polη in TLS, we found that the Rev1 polymerase-dead mutation is synergistic with either Polη mutation or the Polη-interaction mutation in response to UV-induced DNA damage. On the other hand, functional complementation of *polH* cells by Polη relies on its physical interaction with Rev1. Hence, our studies reveal critical interactions between Rev1 and Polη in response to UV damage.

## Introduction

Translesion DNA synthesis (TLS) is a means of DNA damage tolerance (DDT) that allows replication to bypass DNA damage via specialized DNA polymerases with or without associated increase in mutagenesis^1,2^. Mammalian TLS polymerases include Y-family Polη, Polκ, Polι and Rev1; they lack 3′–5′ proofreading exonuclease activity and replicate DNA in a distributive manner^3,4^. In addition, Polζ is a B-family TLS polymerase whose main function is to extend DNA synthesis after initial insertion by a Y-family polymerase opposite the damage site^5,6^. Although a Polζ_2_ complex containing a Rev3 catalytic subunit and a Rev7 regulatory subunit displays TLS polymerase activity in vitro^7^, an active Polζ_4_ in vivo contains two Polδ subunits^8,9^.

The TLS response to UV irradiation has been extensively studied in mammalian cells. UV mainly causes two types of lesions: cyclobutane pyrimidine dimers (CPDs) and pyrimidine (6–4) pyrimidine photoproducts [(6–4)PPs]^10^. Polη bypasses CPDs with high fidelity^11,12^, and defective Polη causes the variant form of the human syndrome xeroderma pigmentosum (XPV) with increased risk of sunlight-induced skin cancer^13,14^. Polη consists of a polymerase core region and a C-terminal domain (CTD), both of which are necessary for its biological functions^15^. The Polη-CTD contains a ubiquitin-binding motif (UBZ), a nuclear localization signal (NLS), two Rev1-interacting (RIR) motifs and two PCNA-interacting (PIP) motifs^3,16,17^. After DNA damage, PCNA is monoubiquitinated at the K164 residue^18^, which signals stalled replication forks^19^. While PIP and UBZ are to enhance interaction with monoubiquitinated PCNA, which is required for Polη to be recruited to the DNA lesions^16^, the role of RIR motifs in TLS remains controversial. It has been reported that Polη is required for the recruitment of Rev1 to the damage site through its RIR motifs, but ectopic expression of the RIR-defective Polη does not affect its ability to protect cells from UV-induced killing and mutagenesis^17^. However, others reported that Rev1 and Polη are independently recruited to the damage site after UV irradiation^20,21^. Furthermore, the PIP motif shares structural similarity with the defined RIR^22^ and indeed can interact with Rev1^23,24^. After UV irradiation, Polη-CTD also promotes Rad18-mediated PCNA monoubiquitination that assists with the recruitment of error-prone TLS polymerases like Polι and Polκ^25^.

It has been well accepted that Rev1 functions as a scaffold for polymerase switch during TLS in response to UV irradiation^26,27^, in which its catalytic activity is dispensable, as the Rev1 polymerase-dead mutation does not confer increased sensitivity to UV-induced killing and mutagenesis^28^. Rev1 can be recruited to the damage site through enhanced affinity for monoubiquitinated PCNA via its PCNA-binding BRCT domain^29,30^ and ubiquitin-binding UBM motifs^31^. The Rev1-CTD contains two separate domains to interact with Y-family polymerases including Polη, Polι and Polκ, and the Rev7 subunit of Polζ^32–35^. We recently attempted to address detailed scaffold roles of Rev1 in response to UV-induced DNA damage and surprisingly found that UV damage tolerance conferred by ectopic expression of Rev1 is dependent on its interaction with Rev7 but independent of Polη interaction^36^. The current study further addressed genetic and physical interactions between Rev1 and Polη, which allowed us to conclude that the Rev1 polymerase can play a backup role in the absence of Polη and that Polη requires its RIR motifs to protect cells from UV-induced DNA damage.

## Results

### Synergistic interaction between Rev1 polymerase and Polη-interaction mutations

We previously reported that UV damage tolerance mediated by PCNA-Ub fusion is dependent on Rev1 but independent of Polη^37^. Since Rev1 interacts with Polη and Rev7 through its Rev1-CTD that can be further divided into two subdomains^34,35^, we screened a large number of reported point mutations in this region^34,35,38,39^ and identified four mutations either specifically affecting Polη but not Rev7 binding (L1170A and V1188A), or disrupting Rev7 but not Polη binding (Y1242A and L1246A). By using an RPA nuclear focus formation assay as an indication of TLS activity after UV irradiation^37,40^, it was found that Rev1-L1170A and Rev1-V1188A protected cells to a level comparable to that of Rev1, while CTD-Y1242A and CTD-L1246A lost Rev1 functions^36^ (Supplementary Fig. S1), which indicates that DDT provided by Rev1 does not require its physical interaction with Polη.

**Figure 1:**
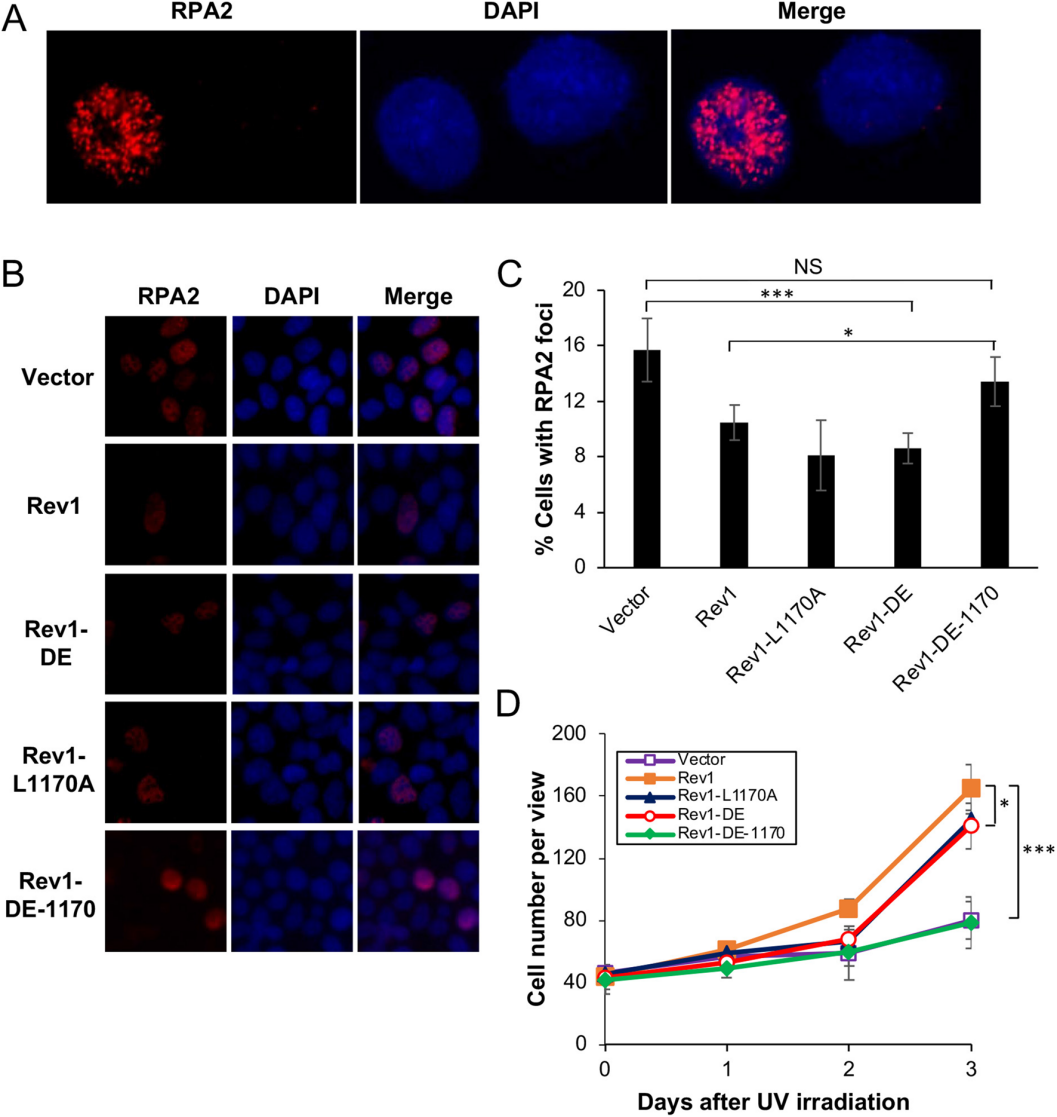
Effects of Rev1 and its mutant derivatives on cellular tolerance to UV irradiation in 293T cells. (**A, B**) Representative images of an RPA nuclear focus formation assay. 293T cells were transfected with plasmids expressing GFP-Rev1 or its mutations. 48 h later, these cells were irradiated by 8 J/m^2^ UV and incubated for 6 h before staining with DAPI or an antibody against RPA2. (**A)** represents typical RPA2-positive (left) and RPA2-negative (right) cells. (**C**) Quantitative analysis of data from (**B)**. (**D**) Effects of Rev1 and its mutations on 293T cell growth in response to UV irradiation. Cells were transfected with plasmids expressing wild-type or indicated *Rev1* point mutations for 2 days before 30 J/m^2^ UV irradiation. (**C**, **D**) Data are means of three independent experiments ± SEM. *, *P* < 0.05; ***, *P* < 0.001; NS, not significant by two-sided Student’s t test.

**Figure 2:**
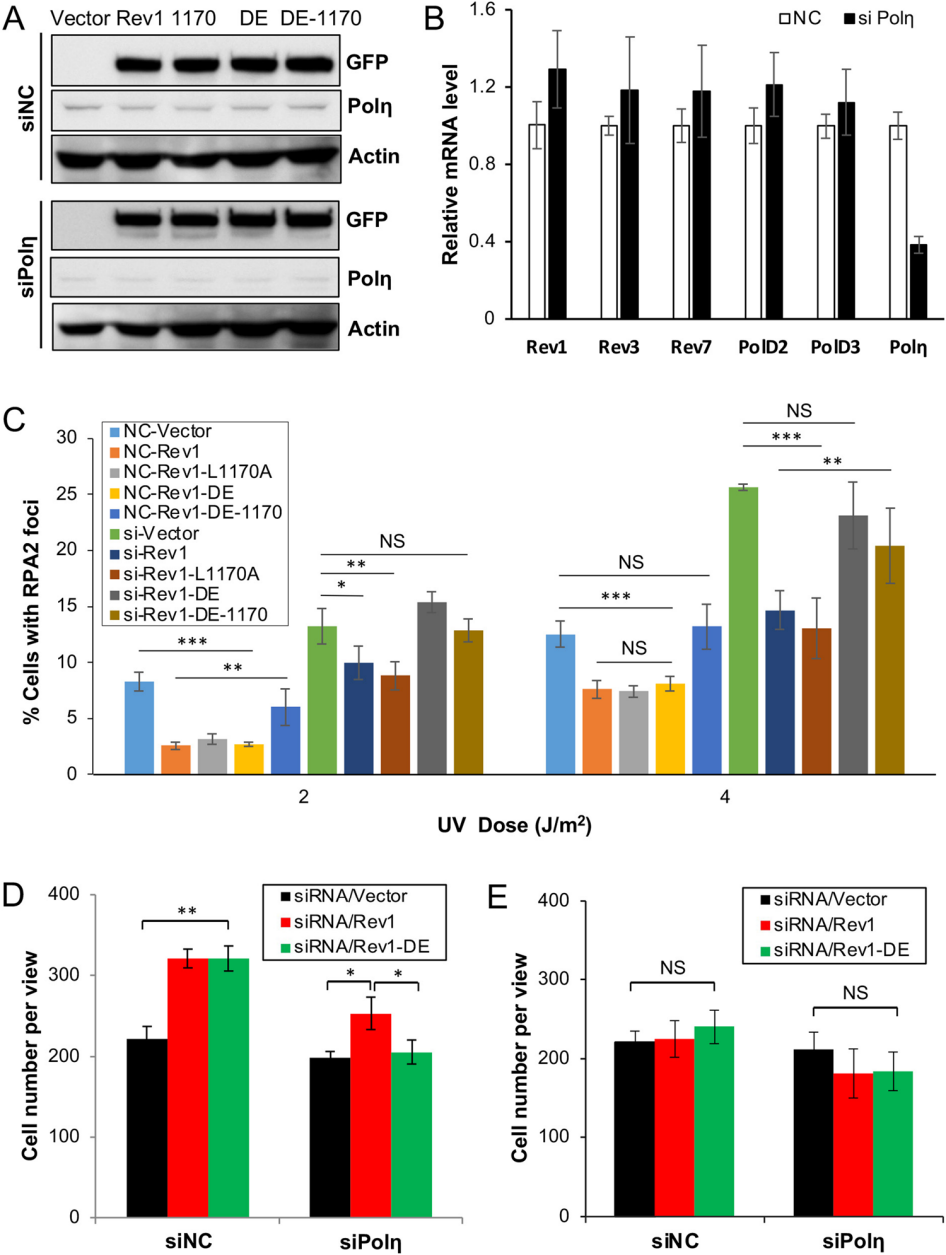
Rev1 and Polη play alternative roles at the insertion step of TLS. (**A**) Western blot analysis of GFP-mRev1 and its mutant transfectants in 293T cells. Cells were transfected with siPolη or non-specific siRNA (siNC). 24 h later, these cells were transfected with plasmids expressing *GFP-mRev1* or its mutant proteins. After 48 h, the transfected cells were harvested, lysed and subjected to western blotting. The two sets of gels were from the same experiment and treated under identical conditions. (**B**) Efficacy of siRNA depletion against Polη in 293T cells as measured by qRT-PCR analysis. (**C**) Effects of *GFP-mRev1* or its mutant expression on UV-induced nuclear RPA2 focus formation in 293T cells with siPolη (si) or non-specific siRNA (NC) treatment. Cells were transfected with siRNA molecules in combination with GFP-mRev1 or its mutants followed by UV irradiation. Immunofluorescence assay was performed 6 h after UV irradiation. (**D, E**) Effects of Polη depletion and ectopic expression of *GFP-mRev1* or *GFP-mRev1-DE* on 293T cell growth with (**D**) or without (**E**) UV irradiation. Cells were transfected with siPolη. 24 h later, these cells were transfected with GFP-mRev1 or its mutant plasmids, incubated for 2 days and irradiated by 20 J/m^2^ UV. After 72 h of incubation, the number of viable cells were counted. Data shown in (**A**, **C**, **D, E)** are means of at least three independent experiments ± SEM. *, *P* < 0.05; **, *P* < 0.01; ***, *P* < 0.001; NS, not significant by two-sided Student’s t test.

The above observations are highly unexpected, as it has been well established in both yeast and mammalian cells that Polη plays a critical role in TLS in response to UV irradiation, and that the Rev1-Polη interaction is critical during this process^3,41^. Based on these observations, we wished to test a hypothesis that in the absence of Rev1-Polη interaction, Rev1 uses its own catalytic activity to initiate TLS in response to UV-induced DNA damage. To this end, we cloned Rev1-DE (Rev1-D568A, E569A, polymerase dead), Rev1-L1170A and the corresponding double mutant Rev1-DE-1170 into pEGFP-C1 as GFP fusions. These plasmids were transfected into 293T cells, with vector pEGFP-C1 as a negative control and Rev1-GFP as a positive control, followed by two functional assays as previously described^37^. For the RPA nuclear focus formation assay, typical RPA2-positive- and negative-cells are illustrated in Fig. 1A. Compared with vector-transfected control, ectopic expression of Rev1 can significantly reduce the percentage of cells with RPA2-positive foci after UV irradiation (Fig. 1B,C), and confer UV damage tolerance (Figs. 1D and S2A). Under the above experimental conditions, expression of either Rev1-DE or Rev1-1170 is sufficient to bring the percentage of RPA2-positive cells to the wild-type level (Fig. 1B,C) and confer near wild-type level UV tolerance (Fig. 1D). In sharp contrast, ectopic expression of the Rev1-DE-1170 double mutant did not confer UV tolerance in either assay in comparison to the control transfected cells (Fig. 1). We ruled out the possibility that the lack of DNA-damage tolerance function of Rev1-DE-1170 was due to altered gene expression or protein stability (Fig. 2A). The above observations collectively allow us to conclude that the Rev1 polymerase and Polη-interaction mutations are synergistic in response to UV-induced DNA damage.

### Rev1 and Polη play alternative roles at the insertion step of TLS in bypassing UV-induced lesions

The Rev1-L1170A mutation likely affects interaction with all three Y-family polymerases, namely Polη, Polι and Polκ^32,33^. Since Polη plays a critical role in cellular response to UV irradiation, we hypothesized that the Rev1 catalytic activity is to back up Polη in the Rev1-L1170A background. To test this hypothesis, we asked whether compromised Polη could replace the Rev1-L1170A mutation. To this end, we depleted the endogenous Polη by siRNA to approximately 16% of the wild-type level (Fig. S3, also see Fig. 4B) while expressing *GFP-REV1* and its various mutations. *GFP-REV1* and its mutants expressed equally well in siPolη cells in comparison to non-specific siNC cells (Figs. 2A and S3), while siPolη specifically reduced the transcript level of the *POLH* gene, but not other relevant genes encoding Rev1 and Polζ subunits (Fig. 2B). After depletion of Polη from 293T cells, the percentage of RPA2-positive cells almost doubled that of the control group, while ectopic expression of Rev1 or Rev1-1170 caused a decrease in RPA2-positive cells to the same extent. Interestingly, expression of Rev1-DE alone provided UV resistance to siNC cells, but failed to protect Polη-depleted cells, in which the percentage of RPA2-positive cells was similar to that of Rev1-DE-1170-transfected cells (Fig. 2C). The above observations indicate that the Polη depletion is epistatic to Rev1-1170A and additive to Rev1-DE. The additive effect between Polη depletion and the Rev1-DE mutation was also seen in a 20 J/m^2^ UV-induced cell survival assay, in which Rev1-DE-transfected cells behave like Rev1 after siNC treatment, but like empty vector after siPolη treatment (Fig. 2D,E). Based on the above observations, we infer that Rev1 and Polη play alternative roles at the insertion step of TLS upon UV irradiation.

### UV damage tolerance conferred by Polη is partially dependent on its interaction with Rev1

**Figure 3:**
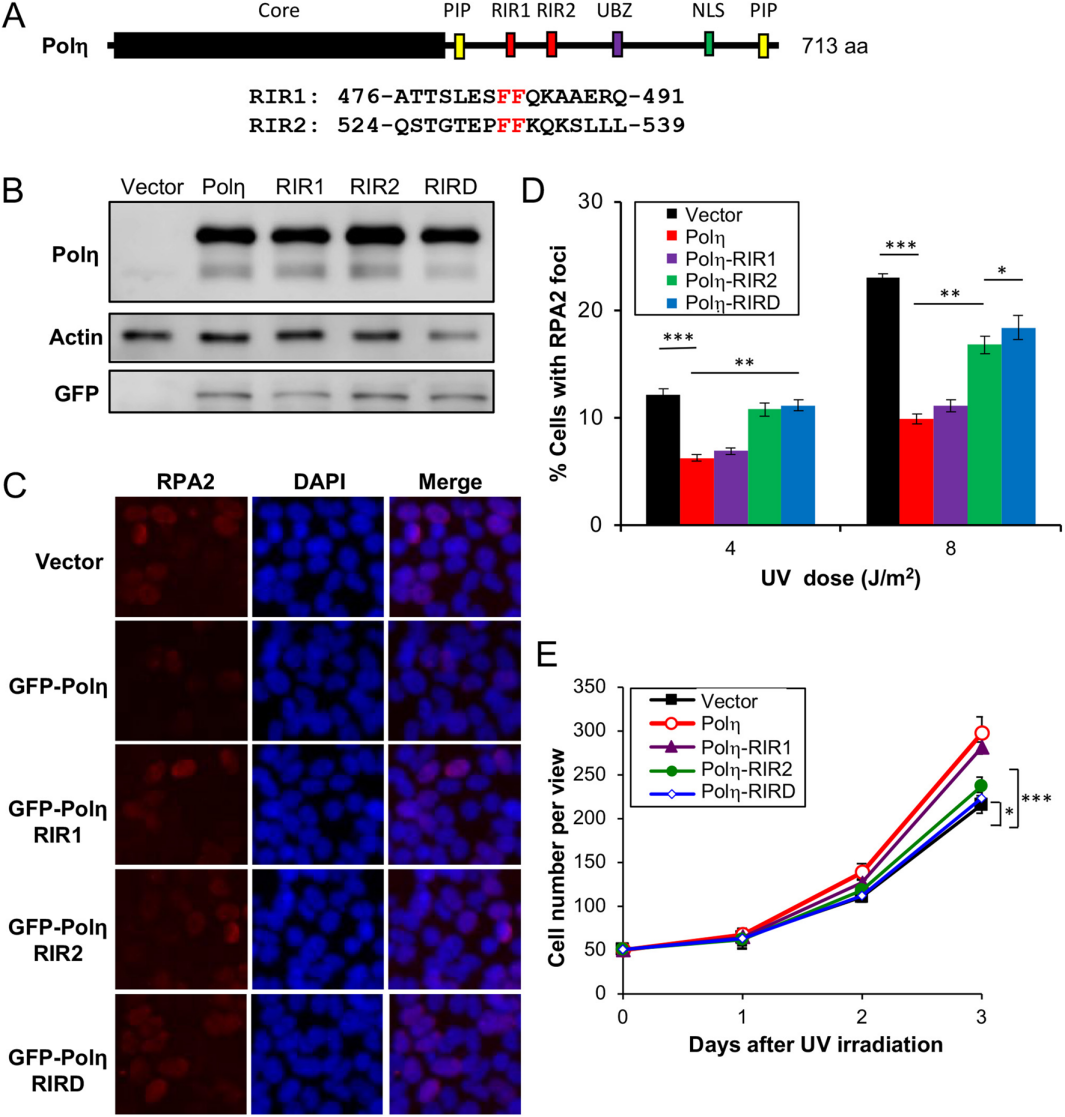
Effects of Polη and its mutation derivatives on cellular tolerance to UV irradiation in T-REx-293 cells. (**A**) Illustration of the Polη structure. Core, the Y-family polymerase catalytic domain; PIP, PCNA-interaction peptide; RIR, Rev1-interaction region; UBZ, Ub zinc-finger; NLS, nuclear localization signal. The RIR1 and RIR2 sequences are aligned with the consensus FF residues in red. (**B**) Western blot analysis of GFP-Polη transfectants. (**C**) Representative images of an RPA nuclear focus formation assay after 8 J/m^2^ UV irradiation. (**D**) Quantitative analysis of the RPA nuclear focus formation assay after 6 h of incubation following UV irradiation. (**E**) Effects of Polη and its mutations on cell growth in response to UV irradiation. Cells were transfected with plasmids expressing wild-type or *POLH* point mutations and incubated for 2 days before 30 J/m^2^ UV irradiation and counting viable cells over time. Results in (**D**, **E**) are means of three independent experiments ± SEM. *, *P* < 0.05; **, *P* < 0.01; ***, *P* < 0.001 by two-sided Student’s t test.

Our observations that the Rev1 interaction with Polη is dispensable appear to contradict a notion of functional importance of physical interaction between Rev1 and other Y-family polymerases during TLS^3,41^. One possibility is that our study was under the Rev1 ectopic expression condition, in which excessive Rev1 is sufficient to provide backup catalytic activity during TLS. It has been previously reported that Polη interacts with Rev1 through residues 369–491^20^ and 509–557^33^, designated as RIR1 and RIR2, respectively (Fig. 3A), and that an FF motif is critical for this interaction^22^. We made RIR1 (Polη-FF483,484AA), RIR2 (Polη-FF531,532AA) and the corresponding double mutation RIRD, and examined their effects on Polη functions. Under our experimental conditions, ectopic expression of *GFP-POLH* and its mutant forms resulted in approximately sixfold more GFP-Polη over endogenous Polη, as judged by western blot analysis (Fig. 3B). GFP-Polη transfection reduced UV-induced RPA2-positive cells (Fig. 3C,D) and protected cells from killing by UV (Figs. 3E and S2B). We then assessed whether the UV damage tolerance conferred by Polη was dependent on its interaction with Rev1.

The Polη-RIR1 mutation seemed to have a moderate effect on the resistance provided by Polη. In contrast, the Polη-RIR2 mutation had a dramatic effect on the Polη function in both RPA2 foci (Fig. 3C,D) and cell survival (Fig. 3E) assays. When T-REx-293 cells were transfected with Polη in which both RIR motifs were mutated, the UV-induced RPA2-positive cells further increased over the level in Polη-RIR2 transfected cells (Fig. 3C,D), indicating that Polη-RIR1 contributes moderately to Rev1 binding. The above observations collectively allow us to conclude that the Polη-Rev1 interaction is mainly through the Polη-RIR2 motif, and that UV damage tolerance conferred by Polη overexpression appears to be partially dependent on its interaction with Rev1.

### *POLH* defective cells were sensitive to UV-induced DNA damage

**Figure 4:**
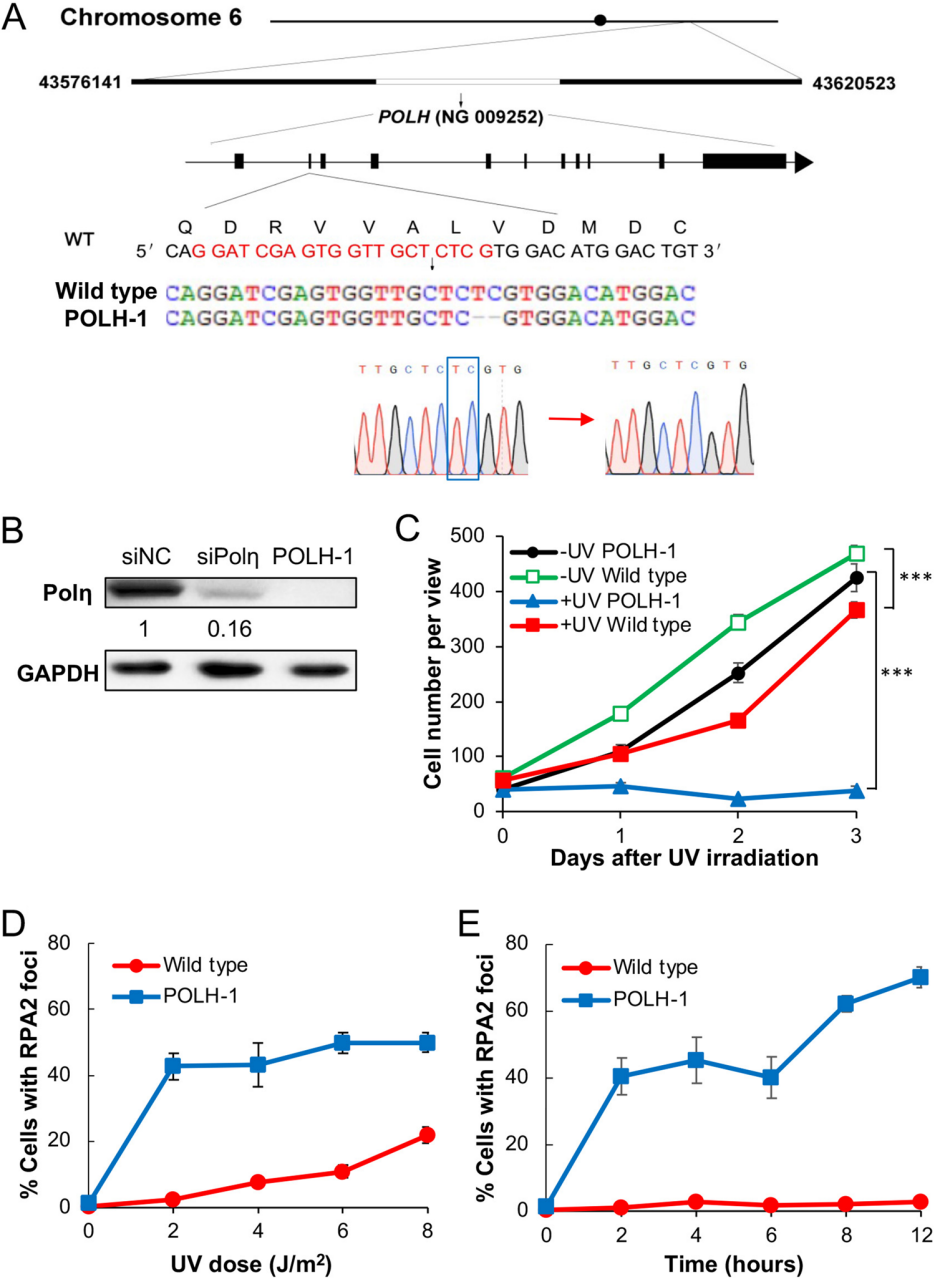
Creation and characterization of an POLH-1 cell line. (**A**) The *POLH* gene location, genomic structure and mutation in the POLH-1 cell line. Nucleotide and encoded amino acid sequences around the guide RNA target (in red) are shown. DNA sequence confirmation of the 2-nt deletion (blue box) in the POLH-1 cells is also illustrated. (**B**) Western blot analysis of Polη in 293T, siPolη-treated and POLH-1 cells. For siPolη depletion, 293T cells were transfected with siPolη molecules and harvested 48 h after treatment. The number indicates the band intensity relative to non-specific siRNA (siNC) treated cells. (**C**) Relative cell growth with or without UV irradiation. 293T and POLH-1 cells were cultured for 2 days followed by 5 J/m^2^ UV irradiation and counting viable cells over time. (**D**) RPA2 focus formation in 293T and POLH-1 cells 4 h after exposure with different UV doses. (**E**) RPA2 focus formation in 293T and POLH-1 cells after 2 J/m^2^ UV irradiation over time. (**C**-**E**) Data are means of three independent experiments ± SEM. ***, *P* < 0.001 by two-sided Student’s t test.

Loss of Polη activity is responsible for the XPV cells^13,14^. To further investigate the role of Rev1 and Polη in TLS, we established *POLH*-inactivated cell lines by knocking out the *XPV/POLH* gene from 293T cells using a CRISPR/Cas9 method^42–44^. One of the cell lines, POLH-1, contains a homozygous 2-bp deletion at the second exon, causing a frameshift mutation (Fig. 4A). A western blot analysis compared endogenous Polη levels in 293T cells, siPolη-treated cells and the isogenic POLH-1 cells. While siPolη treatment reduced cellular Polη by 84%, Polη is undetectable in the POLH-1 cells (Fig. 4B). Compared with the parental 293T cells, POLH-1 cells displayed a relatively normal proliferation rate in the absence of UV irradiation; however, upon 5 J/m^2^ UV irradiation, the POLH-1 cells stopped proliferation over 3 days, whereas the proliferation of 293T cells was only moderately affected (Fig. 4C). 293T cells displayed a characteristic increase in RPA2-positive cells with increasing doses of UV irradiation, reaching approximately 20% at 8 J/m^2^. In contrast, POLH-1 cells dramatically increased RPA2-positive cells to over 40% upon 2 J/m^2^ UV irradiation and did not further increase with increasing doses of UV (Fig. 4D), probably because most cells were dead. 2 J/m^2^ UV irradiation did not significantly induce RPA2 focus formation in 293T cells over time, but drastically induced RPA2 focus formation in POLH-1 cells within 2 h, and the percentage of RPA2-positive cells gradually increased over time (Fig. 4E). The above results confirmed the successful establishment of a *polH* null cell line and demonstrated that POLH-1 cells sustain UV-induced ssDNA as a hallmark of defective TLS.

### Effects of Rev1 and its mutant derivatives on tolerance to UV irradiation in POLH-1 cells

**Figure 5:**
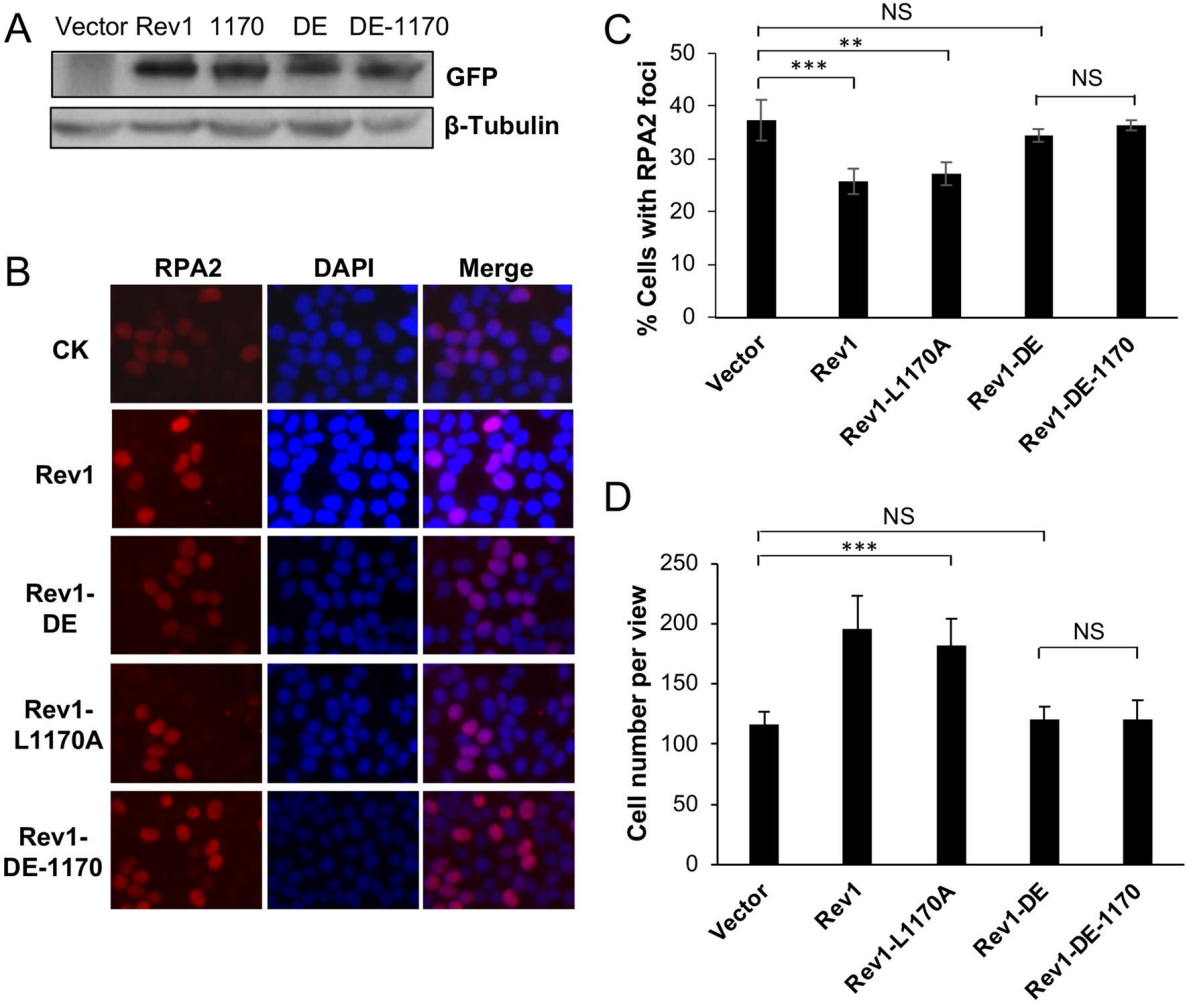
Effects of mRev1 and its mutant derivatives on tolerance to UV irradiation in POLH-1 cells. (**A**) Western blot analysis of transfected GFP-Rev1 and its mutations in POLH-1 cells. (**B**) Representative images of an RPA nuclear focus assay. POLH-1 cells were transfected with plasmids expressing *GFP-Rev1* or its mutations for 2 days followed by 2 J/m^2^ UV irradiation and incubation for 6 h before staining with DAPI or an antibody against RPA2. (**C**) Quantitative analysis of the RPA2 nuclear focus formation. (**D**) Effects of Rev1 and its mutations on POLH-1 cell growth in response to UV irradiation. Cells were transfected with plasmids expressing *GFP-Rev1* or its point mutations for 2 days before 2 J/m^2^ UV irradiation, then these cells were cultured for 48 h, followed by counting viable cells. (**C**, **D**) Results are means of three independent experiments ± SEM. **, *P* < 0.01; ***, *P* < 0.001, NS, not significant by two-sided Student’s t test.

Previously, we hypothesized that the catalytic activity of Rev1 plays a role in nucleotide insertion during TLS either when Rev1 cannot interact with Polη or when the endogenous Polη is reduced. However, the above results are subject to a different interpretation, as they were not obtained from a strict genetic system. With the creation of POLH-1 cells, we were able to critically test our original hypothesis in a clean genetic background. Indeed, in comparison to vector-transfected cells, ectopic expression of *REV1* in POLH-1 (Fig. 5A) could reduce UV-induced RPA2-positive cells (Fig. 5B,C). Under the above experimental conditions, expression of *REV1-L1170A* could rescue POLH-1 cells to the wild-type *REV1* level, whereas expression of *REV1-DE* or the double mutation was no longer able to protect POLH-1 cells (Fig. 5B,C). Similarly, expression of *REV1* or *REV1-L1170A* protected POLH-1 cells from killing by UV irradiation to the same level, while expression of *REV1-DE* or *REV1-DE-1170* had no protective effect (Fig. 5D). These results, together with previous observations^36^, clearly show that when *REV1* is overexpressed, its Rev7 interaction is absolutely required for cellular tolerance against UV damage, while either its catalytic activity or Polη, but not both, is dispensable.

### Effects of Polη and its mutant derivatives on tolerance to UV irradiation in POLH-1 cells

**Figure 6:**
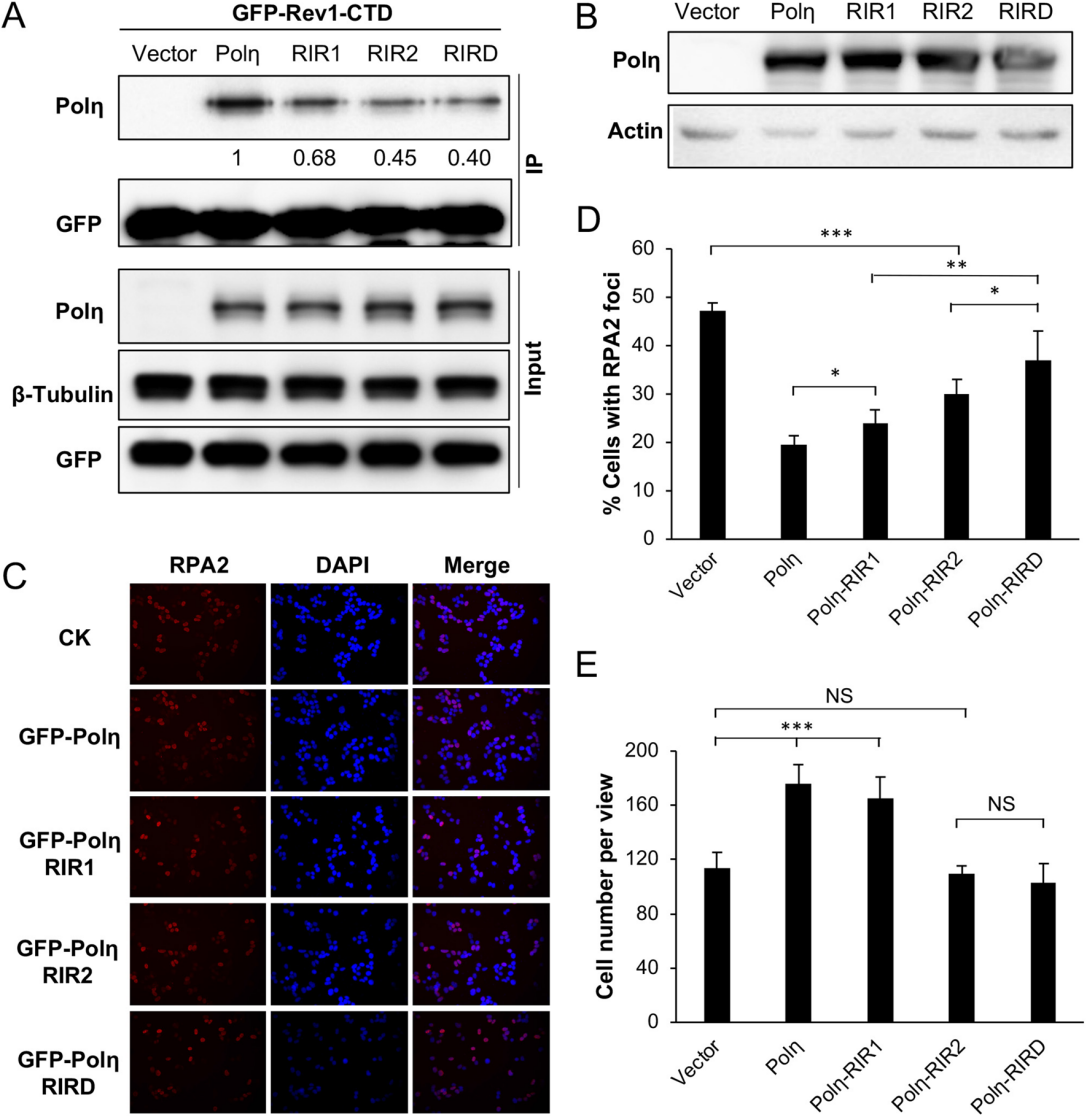
Effects of Polη and its mutant derivatives on UV damage response in POLH-1 cells. (**A**) Co-IP assays to assess the interaction between Rev1 and mutated Polη in POLH-1 cells. (**B**) Western blot analysis of ectopic expression of *POLH* and its mutations in POLH-1 cells. (**C**, **D**) Effects of Polη and its mutations on preventing UV-induced RPA2 nuclear focus formation. POLH-1 cells were transfected with the indicated plasmids and then incubated for 2 days before 4 J/m^2^ UV irradiation, followed by continued culture for 6 h and staining with DAPI or an antibody against RPA2. (**C**) Representative images of the RPA nuclear focus assay. (**D**) Quantitative analysis. (**E**) Effects of Polη and its mutations on POLH-1 cell growth in response to UV irradiation. Cells were transfected with plasmids expressing Polη or its point mutations before 2 J/m^2^ UV irradiation, then these cells were cultured for 48 h, followed by counting viable cells. (**D**, **E**) Results are means of three independent experiments ± SEM. *, *P* < 0.05; ** *P* < 0.01; ***, *P* < 0.001, NS, not significant by two-sided Student’s t test.

Physical interaction between Polη and Rev1 may facilitate TLS; however, it is unclear whether Polη-mediated DDT can bypass the requirement for Rev1. To test whether RIR mutations affect the affinity of Polη for Rev1, we co-transfected POLH-1 cells with GFP-Rev1-CTD and Polη-RIR mutant derivatives followed by co-IP against GFP and western blot analysis against Polη. Figure 6A shows that, compared to wild-type Polη, the Polη-RIR2 mutation had a stronger effect on binding to GFP-Rev1-CTD than the Polη-RIR1 mutation, and the Polη-RIRD double mutation further reduced its affinity for GFP-Rev1-CTD. Hence, RIR2 appears to play a major role in the Polη-Rev1 interaction. The remaining coimmunoprecipitated Polη may come from indirect interactions, as both Rev1 and Polη interact with PCNA and Ub^3,16,29,31^, or from other putative RIR motifs found in Polη^22^.

We transfected plasmids producing GFP-Polη and its RIR mutant forms in POLH-1 cells and monitored their response to UV irradiation. Mutant forms of GFP-Polη did not affect their expression and protein stability in POLH-1 cells under our experimental conditions (Fig. 6B). Expression of *POLH* in POLH-1 reduced UV-induced RPA2-positive cells to a great extent, and expression of *POLH-RIR1* and *POLH-RIR2* mutations partially restored the Polη function in POLH-1 cells, while expression of the *POLH-RIRD* double mutant form of Polη further reduced its rescue ability (Fig. 6C,D). Hence, Polη-RIR1 and Polη-RIR2 mutations appear to be additive in affecting Rev1 interaction, which plays a critical role in TLS. In a cell survival assay, expression of *POLH* restored POLH-1 cell tolerance to 2 J/m^2^ UV and expression of *POLH-RIR1* had a similar effect. In contrast, expression of either *POLH-RIR2* or *POLH-RIRD* failed to rescue POLH-1 cells from killing by UV irradiation (Fig. 6E), indicating that the RIR2 motif plays a critical role in Rev1 interaction and is absolutely required during TLS in response to UV under our experimental conditions. These observations collectively allow us to conclude that interaction with Rev1 is critical for Polη to function in response to UV irradiation.

## Discussion

**Figure 7:**
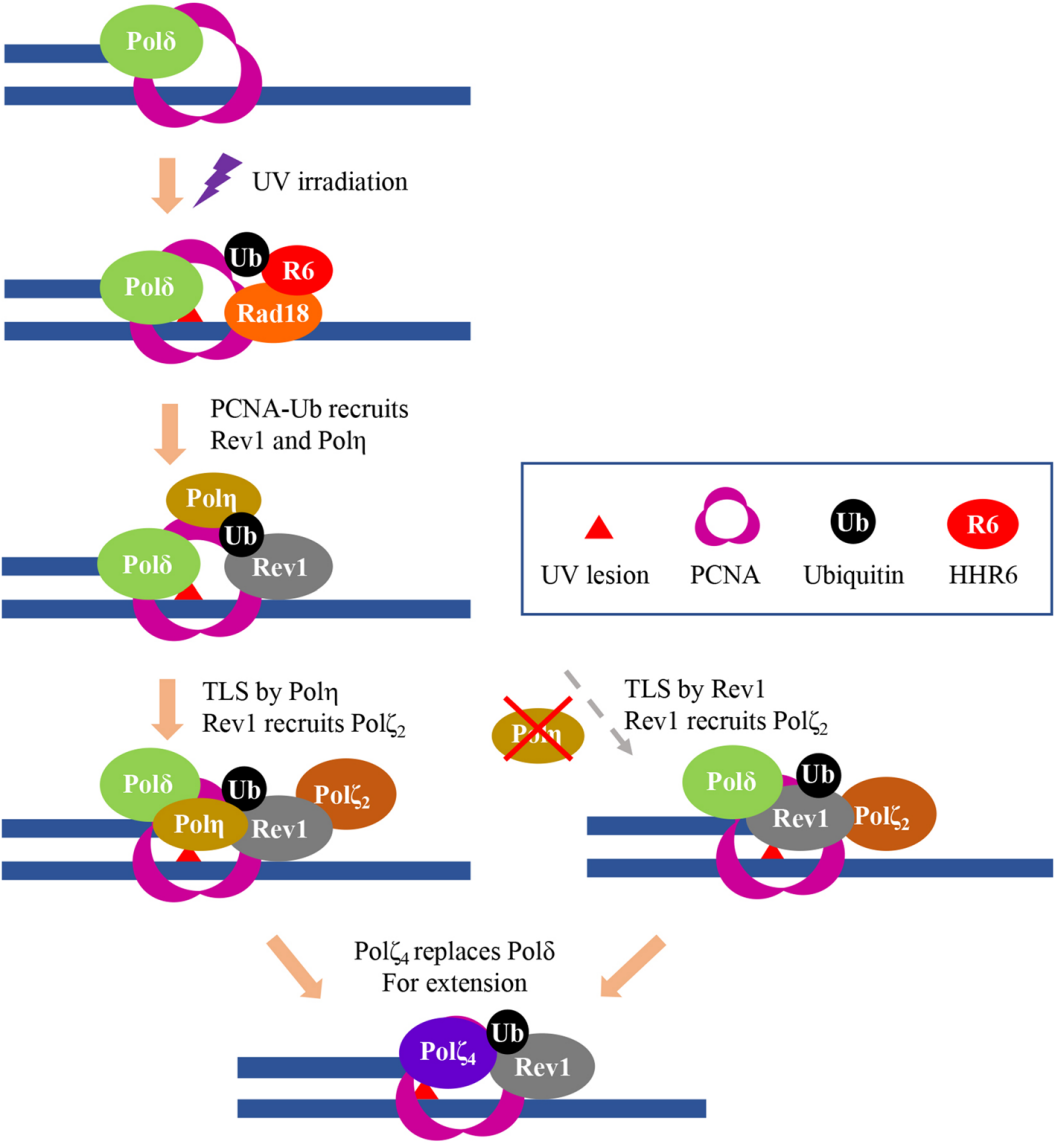
Proposed working model for TLS in response to UV irradiation in mammalian cells. UV irradiation induced DNA damage blocks replicative polymerase like Polδ. The ssDNA along with stalled replication fork recruits the Rad6-Rad18 complex to monoubiquitinate PCNA-K164, which in turn recruits both Polη and Rev1. A default pathway is for Polη to insert nucleotides opposite the lesion, and for Rev1 to recruit Rev7-Rev3 (Polζ_2_) to the damage site to form Polζ_4_ for extension, in which the Polη-Rev1 interaction plays a crucial role. In the absence of Polη, Rev1 can play dual roles in both insertion and Polζ_2_ recruitment, in which its catalytic activity is required.

In mammalian cells, PCNA plays crucial roles in DNA replication and repair^45^. PCNA interacts and travels with all three replicative polymerases during chromosomal DNA replication. When DNA damage stalls the replication fork, PCNA can be ubiquitinated at its K164 residue by Rad6-Rad18, switching to a DDT mode^18^. Monoubiquitinated PCNA enhances affinity for Y-family polymerases^16^ including Polη, Polι, Polκ and Rev1, all of which contain PCNA- and Ub-binding domains^46^. In response to UV irradiation, both Polη and Rev1 are colocalized to the damage sites in the form of nuclear foci^20^. Although subject to debate, our own observations^21,36^ favor a previous report^20^ that they are recruited to the damage site independently from each other, which raises a critical issue: what is the role of Rev1-Polη interaction during TLS? We previously reported that UV damage tolerance conferred by ectopic expression of PCNA-Ub fusion^37^ and Rev1^36^ depends on Rev1 and its physical interaction with Polζ, respectively, but is independent of Polη. Here we show that ectopic expression of Polη can confer additional UV damage tolerance, which requires its RIR domains. Furthermore, expression of Polη can rescue the increased UV sensitivity in Polη-defective cells that mimic the XPV syndrome. This rescue relies on Polη’s physical interaction with Rev1 through RIR motifs. Our observations differ from a previous report^17^ that the Polη-RIR mutations do not affect Polη rescue of XPV cells from killing by UV irradiation, but are consistent with a report^47^ that expression of a polymerase-dead Polη moderately rescues UV sensitivity of Polη-null mouse cells, which depends on its interaction with Rev1. Although both RIR motifs have been reported to mediate interaction with Rev1^20,33^, we found that RIR2 plays a critical role while RIR1 may play a backup role, although RIR1 is critical for the interaction with PolD2^48^. In the absence of RIR motifs, Polη still retains certain physical interaction with Rev1, probably through cryptical RIR motifs found in Polη^22^, although it is insufficient to support the Polη TLS activity. We propose a matchmaker mechanism in which only when cells sense the presence of both Polη and Rev1 at the same damage site through their physical interaction will insertion by Polη and extension by Rev1-mediated Polζ take place to complete the two-step TLS (Fig. 7).

It has been well accepted that in response to UV irradiation, Polη plays a critical catalytic role while Rev1 only plays a scaffold role; the catalytic activity of Rev1 is involved in bypassing abasic sites^49^ and lesions induced by 4-nitroquinoline-1-oxide^50–52^, but is dispensable for lesions induced by UV^28^. However, some studies have indicated direct or indirect roles of Rev1 in bypassing CPD and (6–4)PP^53–55^. Since UV damage tolerance conferred by Rev1 is independent of its physical interaction with Polη^36^, we critically tested a hypothesis that the Rev1’s catalytic activity is responsible for the observed DDT. Firstly, we found a strong synthetic effect between the catalytic and Polη-binding mutations in Rev1. While either the Rev1-DE or Rev1-L1170A mutation can provide DDT to near the wild-type Rev1 level, the double mutation behaves like a null mutation. Secondly, we ruled out a possibility that other TLS polymerases are responsible for the effect by experimental depletion of Polη, and found that once Polη is depleted, the Rev1-L1170 mutation still behaves like wild-type Rev1, while the Rev1-DE mutation is no longer able to provide DDT. Finally, using a newly created POLH-1 cell line, we showed that the *polh* is additive with the Rev1-DE mutation but epistatic to the Rev1-L1170A mutation. These observations collectively support a notion that Polη plays a frontline role in TLS insertion across from UV-induced lesions and that Rev1 is required for its non-catalytic role, probably through recruiting Polζ_2_. However, in the absence of Polη, Rev1 can insert nucleotide(s) across from UV-induced lesions as well as recruit Polζ_2_ to the damage site (Fig. 7). A major critique for the above model is that it is based on ectopic expression of Rev1, which may not occur in untransfected cells. Interestingly, a synergistic interaction between Rev1-DE and Polη in response to UV irradiation is recently reported in budding yeast^56^, which lends strong support to the working model. Rev1 may also facilitate the assembly of Polζ_4_ by physical interaction with PolD3^57^, which is likely an active form for TLS extension^58^ (Fig. 7). Rev1 is a template-dependent dCMP transferase^51,59^. Since UV-induced lesions are almost exclusively on pyrimidines^60^, the dCMP insertion by Rev1 is expected to cause transversion mutations. Fortunately Rev1 has very limited catalytic activity toward major UV-induced lesions^52^, and it must be kept at bay until needed.

## Methods

### Plasmids and plasmid construction

The open reading frames of *POLH* and *mRev1* were cloned in pEGFP-C1 (BD Biosciences Clontech). Polη point mutants were created with the following primers: RIR1-F: 5′-ACCACGTCTGGAATCAGCCCAAAGCTGCAGAAAGG-3′; RIR1-R: 5′-CCTTTCTGCAGCTTTGGGGGGCTGATTCCAGACGTGGT-3′; RIR2-F: 5′-AGTACAGGAACTGAGCCCGCTAAGCAAAAGTCTGCTT-3′; RIR2-R: 5′-AAGCAGACTTTTCT TG TAGCGGGCTCAGTTCCTGTACT-3′. Rev1 point mutants were created with the following primers: Rev1-L1170A-F: 5′-AGTGATGTGAAGACCTTGGCCAAAGAGTGGATCACTACT-3′; Rev1-L1170A-R: 5′-AGTAGTGATCCACTCTTTGGCCAAGGTCTTCACATCACT-3′; Rev1-DE-F: 5′-ATCGAGGCTGTCAGCTGCGCTGCAGCACTGATTGACGTCACG-3′; Rev1-DE-R: 5′-CGTGACGTCAATCAGTGCTGCAGCGCAGCTGACAGCCTCGAT-3′.

For the co-IP assay, DNA sequences corresponding to *Rev1-CTD* (residues 1150–1249) were PCR-amplified and cloned into pEGFP-C1 to produce EGFP-fused proteins. *POLH* and its mutant coding sequences were cloned into vector pcDNA4/TO (Invitrogen).

### Cell culture and reagents

Human T-REx293 and 293T cells were purchased from Invrogen, and POLH-1 cells were created from 293T cells in this study. Cells were cultured in a DMEM medium supplemented with 10% fetal bovine serum at 37 °C in the presence of 5% CO_2_. For transient transfection experiments, T-REx293 and 293T cells were transfected with indicated plasmids by using PEI (Polyethylenimine, Linear, MW 25,000, Polysciences) following the manufacturer’s protocols. In order to enrich transfected cells over 50%, G418 was added to a final concentration of 200 µg/mL 24 h after transfection. POLH-1 cells were transfected with indicated plasmids by using Lipofectamine 2000 (Invitrogen) to achieve 40–50% transfection efficiency without subsequent antibiotic selection.

### Generation of *polh* cell lines from 293T cells

CRISPR/Cas9-mediated gene targeting was performed by using a Genloci CRISPR/Cas9 kit with EGFP + Puro^r^ (GP0129, Genloci) as described (Protocol No. PT161117-1). Briefly, the *POLH*-targeting double-strand oligonucleotide, made by annealing Polη-F: 5′-caccGGATCGAGTGGTTGCTCTCG-3′ and Polη-R: 5′- aaacCGAGAGCAACCACTCGATCC-3′, was cloned into plasmid pGK1.2. The resulting plasmid was used to transfect 293T cells, and the transfectants were cultured in a DMEM medium supplemented with 10% fetal bovine serum. Puromycin (Sigma) was used to a final concentration of 1 µg/mL to select transfectants over 14 days, and puromycin-resistant clones were transferred to a 96-well plate for expansion and screening of *POLH* knockouts. The targeted clones were confirmed by genomic PCR with primers Polη Primer-F: 5′-CCATGCTCCCATGCTCATGGTAACTC-3′ and Polη Primer-R: 5′-CCTGCCACAGTGCCACTGTGTTACC-3′, and the PCR products were sent for sequencing.

### RNA interference

The depletion of endogenous Polη in 293T cells was performed as previously described^37^. The *POLH* gene-specific target sequence (siPolη) 5′-CTGGTTGTGAGCATTCGTGTA-3′ and the scrambled siRNA (siNC) were purchased from Shanghai GenePharm. The suppression efficacy was assessed by quantitative RT-PCR (qRT-PCR) and/or western blotting 48 h after siRNA transfection. Primers used for qRT-PCR include RT-Polη-F: 5′-GCAGCCATAGAGAGGGAGAC-3′, RT-Polη-R: 5′-CTCCTTAATGTCACGCACGAT-3′, hRev1-F: 5′-ACCGAAGAGGAGCACAAAGA-3′, hRev1-R: 5′-CCATTCCATTTCCCTGAAGA-3′, hRev3-F: 5′-AGTAAATGTCGGAGCCAAC-3′, hRev3-R: 5′-CTGGGCAGTTCAGAGAAACA-3′, hRev7-F: 5′-TGGCTGTGCATCTCATCCTCT-3′, hRev7-R: 5′-GCGGTGCTCTTTATCCAAAATCA-3′, hPolD2-F: 5′-CCATCAGCCAACAATGCCAC-3′, hPolD2-R: 5′-CTAGCCGGAAGGGTTGTGA-3′, hPolD3-F: 5′-GAGTTCGTCACGGACCAAAAC-3′, and hPolD3-R: 5′-GCCAGACACCAAGTAGGTAAC-3′.

### Cell survival assay

The 293T or POLH-1 cells were cultured in 6-cm culture dishes, and then transfected with plasmids carrying the gene of interest. After incubation for 48 h, the cells were irradiated with UV at the given doses, cultured for up to 3 days and then fixed with 4% formaldehyde. The fixed cultures were stained with DAPI and photographs were taken from random fields in dish for cell counting. Cells with round and intact nuclei were counted as viable cells, and images were acquired using the CCD RoHs (Q26053) as previously described^37^. At least 2000 cells were counted for each treatment.

### RPA nuclear focus formation assay

Cultured cells were seeded on poly-lysine-coated cover slips, rinsed once with ice-cold PBS (2.25 g Na_2_HPO_4_, 8 g NaCl, 0.2 g KH_2_PO_4_, 2 g KCl dissolved in 1 L ddH_2_O), treated with 0.4% NP-40 in PBS for 20 min on ice, and then fixed with 4% paraformaldehyde for 15 min. The fixed cells were rinsed 3 times with PBS, treated with methanol for 5 min, and then rinsed 4 times with PBST for 5 min each time. After incubation with 5% FBS in PBST for 45 min, cells were incubated with mouse anti-Replication protein A2 (RPA2) antibody (Abeam, Ab2175, 1:1000) overnight. The cells were washed four times with PBST, incubated with Alexa Flour 546 goat anti-mouse secondary antibody (Invitrogen, A11030, 1:1000) and 1.5 µg/mL 4′,6-diamidino-2-phenylindole (DAPI) at room temperature for 1 h, and finally washed 4 times with PBST again. For quantitative analysis of UV-induced RPA2 focus formation, the 293T or POLH-1 cells transfected with corresponding plasmids were treated with UV. Images were taken with the same exposure time. Microscopy was performed with an inverted Olympus 10*22 microscope equipped with a 40 × immersion lens, and images were acquired using the CCD RoHs (Q26053) as previously described^37^. At least 1000 cells were counted for each treatment.

### Co-immunoprecipitation (Co-IP) and western blotting

To measure the expression levels of GFP*-*Rev1 and Polη or their mutant derivatives, 293T cells were transfected with the plasmids. 2 days later, cell lysates were harvested, boiled before SDS-PAGE, and detected by indicated antibodies against Polη (Abcam, ab17725) and the GFP Tag (Abmart, 7G9). The following reference protein antibodies were from Lifetech: GAPDH (GA331), β-Actin (GA321) and β-tubulin (GA311).

For the co-IP assay, the POLH-1 cells transfected with GFP-Rev1-CTD and Polη RIR mutants were harvested and immunoprecipitated with GFP-Trap A (ChromoTek, gta-20) overnight. The input and the immunoprecipitated proteins were separated by SDS-PAGE and proteins of interest were detected by indicated antibodies against Polη, β-tubulin and GFP.

## Supplementary Information

Supplementary Information 1.

Supplementary Information 2.

## References

[1] Shachar S (2009). Two-polymerase mechanisms dictate error-free and error-prone translesion DNA synthesis in mammals. EMBO J..

[2] Zhang W, Qin Z, Zhang X, Xiao W (2011). Roles of sequential ubiquitination of PCNA in DNA-damage tolerance. FEBS Lett..

[3] Yang W, Woodgate R (2007). What a difference a decade makes: insights into translesion DNA synthesis. Proc. Natl. Acad. Sci. U.S.A..

[4] Andersen PL, Xu F, Xiao W (2008). Eukaryotic DNA damage tolerance and translesion synthesis through covalent modifications of PCNA. Cell Res..

[5] Johnson RE, Washington MT, Haracska L, Prakash S, Prakash L (2000). Eukaryotic polymerases iota and zeta act sequentially to bypass DNA lesions. Nature.

[6] Prakash S, Prakash L (2002). Translesion DNA synthesis in eukaryotes: a one- or two-polymerase affair. Genes Dev..

[7] Nelson JR, Lawrence CW, Hinkle DC (1996). Thymine-thymine dimer bypass by yeast DNA polymerase zeta. Science.

[8] Johnson RE, Prakash L, Prakash S (2012). Pol31 and Pol32 subunits of yeast DNA polymerase delta are also essential subunits of DNA polymerase zeta. Proc. Natl. Acad. Sci. U.S.A..

[9] Makarova AV, Stodola JL, Burgers PM (2012). A four-subunit DNA polymerase zeta complex containing Pol delta accessory subunits is essential for PCNA-mediated mutagenesis. Nucleic Acids Res..

[10] Sinha RP, Hader DP (2015). Physiological aspects of UV-excitation of DNA. Top. Curr. Chem..

[11] Johnson RE, Prakash S, Prakash L (1999). Efficient bypass of a thymine-thymine dimer by yeast DNA polymerase, Poleta. Science.

[12] Biertumpfel C (2010). Structure and mechanism of human DNA polymerase eta. Nature.

[13] Johnson RE, Kondratick CM, Prakash S, Prakash L (1999). hRAD30 mutations in the variant form of xeroderma pigmentosum. Science.

[14] Masutani C (1999). The XPV (xeroderma pigmentosum variant) gene encodes human DNA polymerase eta. Nature.

[15] Prakash S, Johnson RE, Prakash L (2005). Eukaryotic translesion synthesis DNA polymerases: specificity of structure and function. Annu. Rev. Biochem..

[16] Bienko M (2005). Ubiquitin-binding domains in Y-family polymerases regulate translesion synthesis. Science.

[17] Akagi J (2009). Interaction with DNA polymerase eta is required for nuclear accumulation of REV1 and suppression of spontaneous mutations in human cells. DNA Repair (Amst.).

[18] Hoege C, Pfander B, Moldovan GL, Pyrowolakis G, Jentsch S (2002). RAD6-dependent DNA repair is linked to modification of PCNA by ubiquitin and SUMO. Nature.

[19] Pastushok L, Xiao W (2004). DNA postreplication repair modulated by ubiquitination and sumoylation. Adv. Protein Chem..

[20] Tissier A (2004). Co-localization in replication foci and interaction of human Y-family members, DNA polymerase pol eta and REVl protein. DNA Repair (Amst.).

[21] Andersen PL, Xu F, Ziola B, McGregor WG, Xiao W (2011). Sequential assembly of translesion DNA polymerases at UV-induced DNA damage sites. Mol. Biol Cell.

[22] Ohashi E (2009). Identification of a novel REV1-interacting motif necessary for DNA polymerase kappa function. Genes Cells.

[23] Boehm EM (2016). The proliferating cell nuclear antigen (PCNA)-interacting protein (PIP) motif of DNA polymerase eta mediates its interaction with the C-terminal domain of Rev1. J. Biol. Chem..

[24] Boehm EM, Washington MT (2016). RIP to the PIP: PCNA-binding motif no longer considered specific: PIP motifs and other related sequences are not distinct entities and can bind multiple proteins involved in genome maintenance. BioEssays.

[25] Durando M, Tateishi S, Vaziri C (2013). A non-catalytic role of DNA polymerase eta in recruiting Rad18 and promoting PCNA monoubiquitination at stalled replication forks. Nucleic Acids Res..

[26] Nelson JR, Gibbs PE, Nowicka AM, Hinkle DC, Lawrence CW (2000). Evidence for a second function for Saccharomyces cerevisiae Rev1p. Mol. Microbiol..

[27] Friedberg EC, Lehmann AR, Fuchs RP (2005). Trading places: how do DNA polymerases switch during translesion DNA synthesis?. Mol. Cell.

[28] Zhou Y, Wang J, Zhang Y, Wang Z (2010). The catalytic function of the Rev1 dCMP transferase is required in a lesion-specific manner for translesion synthesis and base damage-induced mutagenesis. Nucleic Acids Res..

[29] Guo C (2006). REV1 protein interacts with PCNA: significance of the REV1 BRCT domain in vitro and in vivo. Mol. Cell.

[30] Otsuka C, Kunitomi N, Iwai S, Loakes D, Negishi K (2005). Roles of the polymerase and BRCT domains of Rev1 protein in translesion DNA synthesis in yeast in vivo. Mutat. Res..

[31] Guo C (2006). Ubiquitin-binding motifs in REV1 protein are required for its role in the tolerance of DNA damage. Mol. Cell Biol..

[32] Guo C (2003). Mouse Rev1 protein interacts with multiple DNA polymerases involved in translesion DNA synthesis. EMBO J..

[33] Ohashi E (2004). Interaction of hREV1 with three human Y-family DNA polymerases. Genes Cells.

[34] Wojtaszek J (2012). Multifaceted recognition of vertebrate Rev1 by translesion polymerases zeta and kappa. J. Biol. Chem..

[35] Xie W, Yang X, Xu M, Jiang T (2012). Structural insights into the assembly of human translesion polymerase complexes. Protein Cell.

[36] Niu X (2019). Rev1 plays central roles in mammalian DNA-damage tolerance in response to UV irradiation. FEBS J..

[37] Qin Z (2013). DNA-damage tolerance mediated by PCNA*Ub fusions in human cells is dependent on Rev1 but not Poleta. Nucleic Acids Res..

[38] Pozhidaeva A (2012). NMR structure and dynamics of the C-terminal domain from human Rev1 and its complex with Rev1 interacting region of DNA polymerase eta. Biochemistry.

[39] Wojtaszek J (2012). Structural basis of Rev1-mediated assembly of a quaternary vertebrate translesion polymerase complex consisting of Rev1, heterodimeric polymerase (Pol) zeta, and Pol kappa. J. Biol. Chem..

[40] Diamant N (2012). DNA damage bypass operates in the S and G2 phases of the cell cycle and exhibits differential mutagenicity. Nucleic Acids Res..

[41] Lehmann AR (2007). Translesion synthesis: Y-family polymerases and the polymerase switch. DNA Repair (Amst.).

[42] Cong L (2013). Multiplex genome engineering using CRISPR/Cas systems. Science.

[43] Ran FA (2013). Genome engineering using the CRISPR-Cas9 system. Nat. Protoc..

[44] Yang, L., Yang, J. L., Byrne, S., Pan, J. & Church, G. M. CRISPR/Cas9-Directed Genome Editing of Cultured Cells. *Curr Protoc Mol Biol* **107**, 31 31 31–17, doi:10.1002/0471142727.mb3101s107 (2014). 10.1002/0471142727.mb3101s107 24984853

[45] Moldovan GL, Pfander B, Jentsch S (2007). PCNA, the maestro of the replication fork. Cell.

[46] Waters LS (2009). Eukaryotic translesion polymerases and their roles and regulation in DNA damage tolerance. Microbiol. Mol. Biol. Rev..

[47] Ito W (2012). Stalled Poleta at its cognate substrate initiates an alternative translesion synthesis pathway via interaction with REV1. Genes Cells: Devot. Mol. Cell. Mech..

[48] Baldeck N (2015). FF483-484 motif of human Poleta mediates its interaction with the POLD2 subunit of Poldelta and contributes to DNA damage tolerance. Nucleic Acids Res..

[49] Kim N, Mudrak SV, Jinks-Robertson S (2011). The dCMP transferase activity of yeast Rev1 is biologically relevant during the bypass of endogenously generated AP sites. DNA Repair (Amst.).

[50] Wiltrout ME, Walker GC (2011). The DNA polymerase activity of Saccharomyces cerevisiae Rev1 is biologically significant. Genetics.

[51] Nair DT, Johnson RE, Prakash L, Prakash S, Aggarwal AK (2005). Rev1 employs a novel mechanism of DNA synthesis using a protein template. Science.

[52] Zhang Y (2002). Response of human REV1 to different DNA damage: preferential dCMP insertion opposite the lesion. Nucleic Acids Res..

[53] Temviriyanukul P (2012). Temporally distinct translesion synthesis pathways for ultraviolet light-induced photoproducts in the mammalian genome. DNA Repair (Amst.).

[54] Gibbs PE, McDonald J, Woodgate R, Lawrence CW (2005). The relative roles in vivo of Saccharomyces cerevisiae Pol eta, Pol zeta, Rev1 protein and Pol32 in the bypass and mutation induction of an abasic site, T-T (6–4) photoadduct and T-T cis-syn cyclobutane dimer. Genetics.

[55] Quinet A (2016). Translesion synthesis mechanisms depend on the nature of DNA damage in UV-irradiated human cells. Nucleic Acids Res..

[56] Wang Z, Xiao W (2020). Distinct requirements for budding yeast Rev1 and Poleta in translesion DNA synthesis across different types of DNA damage. Curr. Genet..

[57] Pustovalova Y (2016). Interaction between the Rev1 C-Terminal domain and the PolD3 subunit of Polzeta suggests a mechanism of polymerase exchange upon Rev1/Polzeta-dependent translesion synthesis. Biochemistry.

[58] Lee YS, Gregory MT, Yang W (2014). Human Pol zeta purified with accessory subunits is active in translesion DNA synthesis and complements Pol eta in cisplatin bypass. Proc. Natl. Acad. Sci. USA.

[59] Nelson JR, Lawrence CW, Hinkle DC (1996). Deoxycytidyl transferase activity of yeast REV1 protein. Nature.

[60] Bastien N, Therrien JP, Drouin R (2013). Cytosine containing dipyrimidine sites can be hotspots of cyclobutane pyrimidine dimer formation after UVB exposure. Photochem. Photobiol. Sci..

