# Supplementary material for: Genetic and physical interactions between Polη and Rev1 in response to UV-induced DNA damage in mammalian cells
Source: Sci Rep. 2021 Nov 1;11:21364. doi: 10.1038/s41598-021-00878-3 (PMC8560953; doi:10.1038/s41598-021-00878-3)
Supplement: Supplementary file 1 — Supplementary Information 1. [file 41598_2021_878_MOESM1_ESM.pdf]

Supplementary figures

Figure legends

Figure S1. UV-damage tolerance conferred by Rev1 requires its physical interaction with Rev7 but not Polη. T-REx-293 cells were transfected with plasmids expressing *GFP-Rev1* or its CTD mutant derivatives. 20 hours later, cells were exposed to 8 J/m^2 UV and incubated for 6 hours before being fixed with paraformaldehyde for the RPA nuclear focus formation assay. (**A**) Representative images of cells stained with DAPI or antibodies against RPA2. (**B**) Quantitative analysis of the percentage of cells with RPA2 foci. Data are means of three independent experiments ± SEM. ***, $P<0.001$; NS, not significant by two-sided Student's t test. Results were adapted from Supplementary Fig. S1 in Niu et al. (*FEBS J.* **286**, 2711-2725, 2019).

Figure S2. Effects of GFP-Rev1 and GFP-Polη transient transfection on cell growth. (**A**) GFP-Rev1 transfection. (**B**) GFP-Polη transfection. 293T cell were transfected with plasmids producing GFP-Rev1 and GFP-Polη or their mutant derivatives. Viable cells were counted over a period of 3 days. Results are means of three independent experiments ± SEM.

Figure S3. Efficacy of Polη depletion by siPolη in GFP-Rev1 transfected cells. 293T cells were first transfected with either siPolη or non-specific siRNA (siNC). After 24-hour incubation, they were transfected with plasmids expressing *GFP-mRev1* or its mutant proteins. After 48 hours, the transfected cells were harvested, lysed, and subjected to western blot analysis.

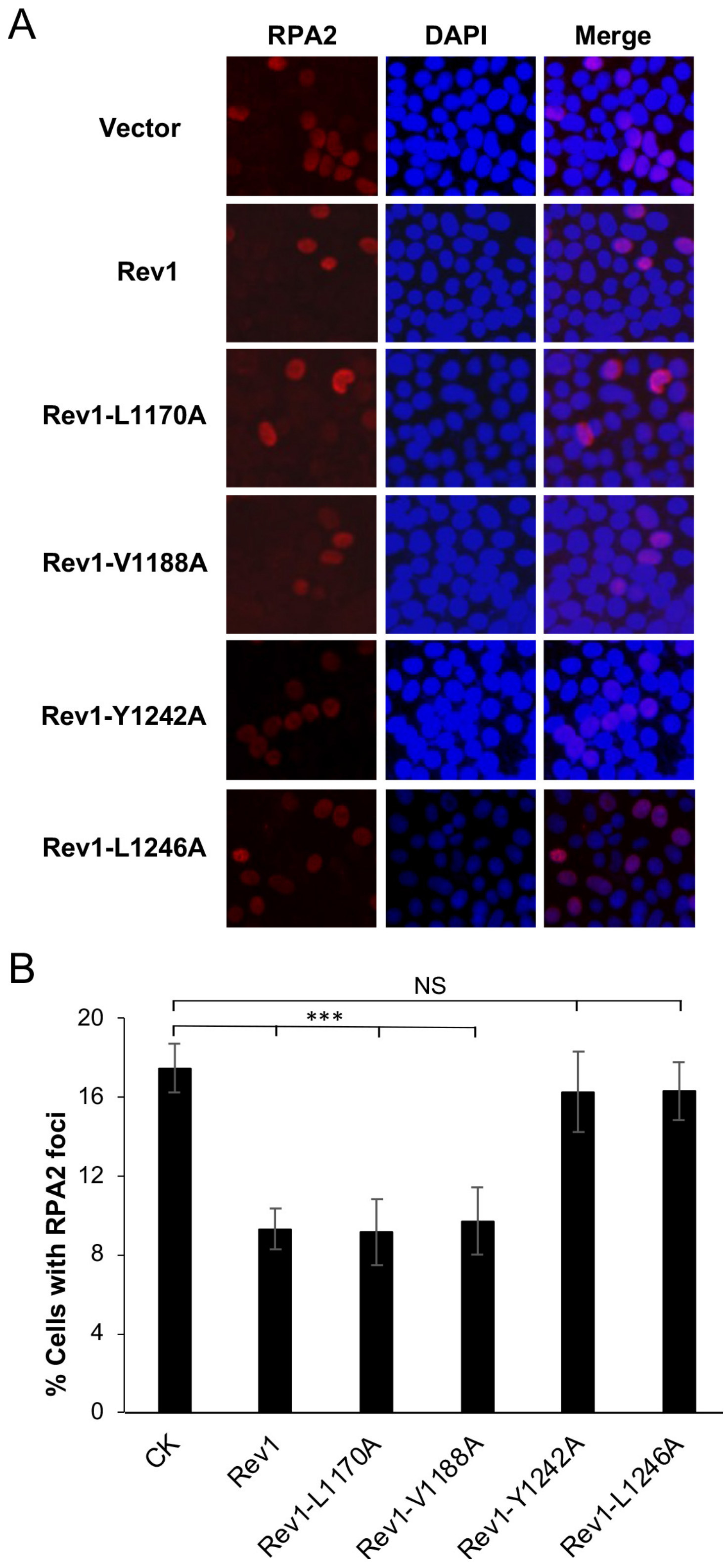
A
RPA2
DAPI
Merge
Vector
Rev1
Rev1-L1170A
Rev1-V1188A
Rev1-Y1242A
Rev1-L1246A
B
NS
***
% Cells with RPA2 foci
20
16
12
8
4
0
CK
Rev1
Rev1-L1170A
Rev1-V1188A
Rev1-Y1242A
Rev1-L1246A

Figure S1

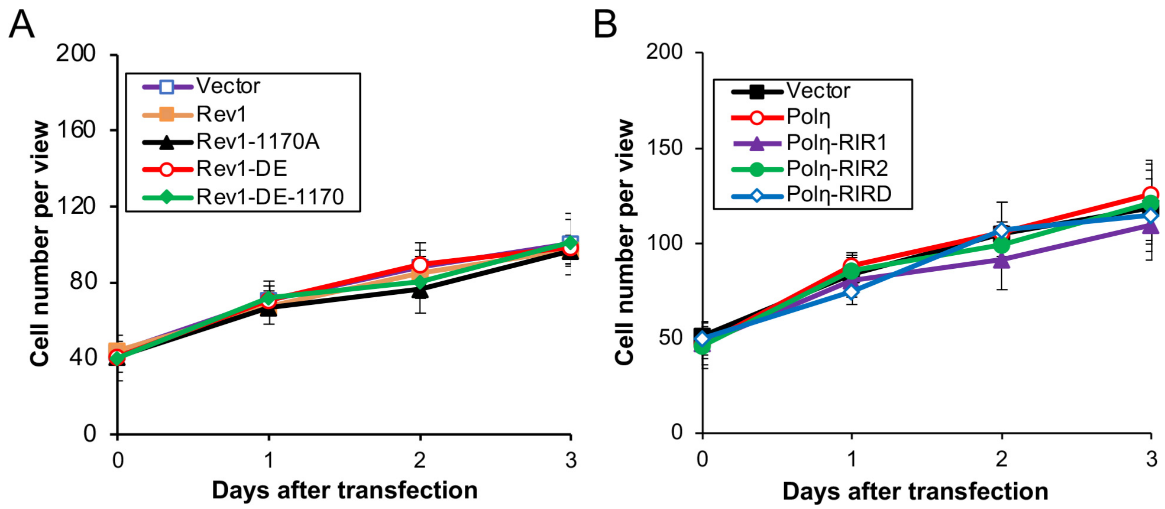
A
200
160
120
80
40
0
Cell number per view
Vector
Rev1
Rev1-1170A
Rev1-DE
Rev1-DE-1170
0
1
2
3
Days after transfection
B
200
150
100
50
0
Cell number per view
Vector
Polη
Polη-RIR1
Polη-RIR2
Polη-RIRD
0
1
2
3
Days after transfection

Figure S2

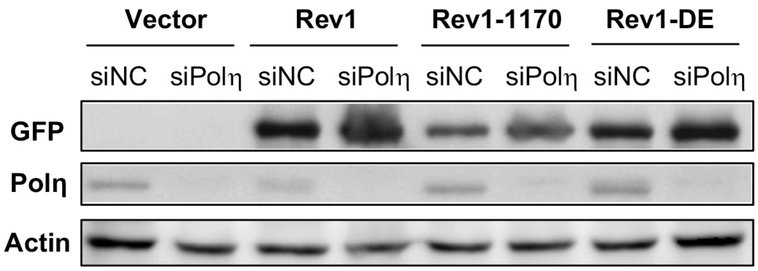
Vector
Rev1
Rev1-1170
Rev1-DE
siNC
siPolη
siNC
siPolη
siNC
siPolη
siNC
siPolη
GFP
Polη
Actin

Figure S3
