# Supplementary figures and images for: Genetic and physical interactions between Polη and Rev1 in response to UV-induced DNA damage in mammalian cells
Source: Sci Rep. 2021 Nov 1;11:21364. doi: 10.1038/s41598-021-00878-3 (PMC8560953; doi:10.1038/s41598-021-00878-3)

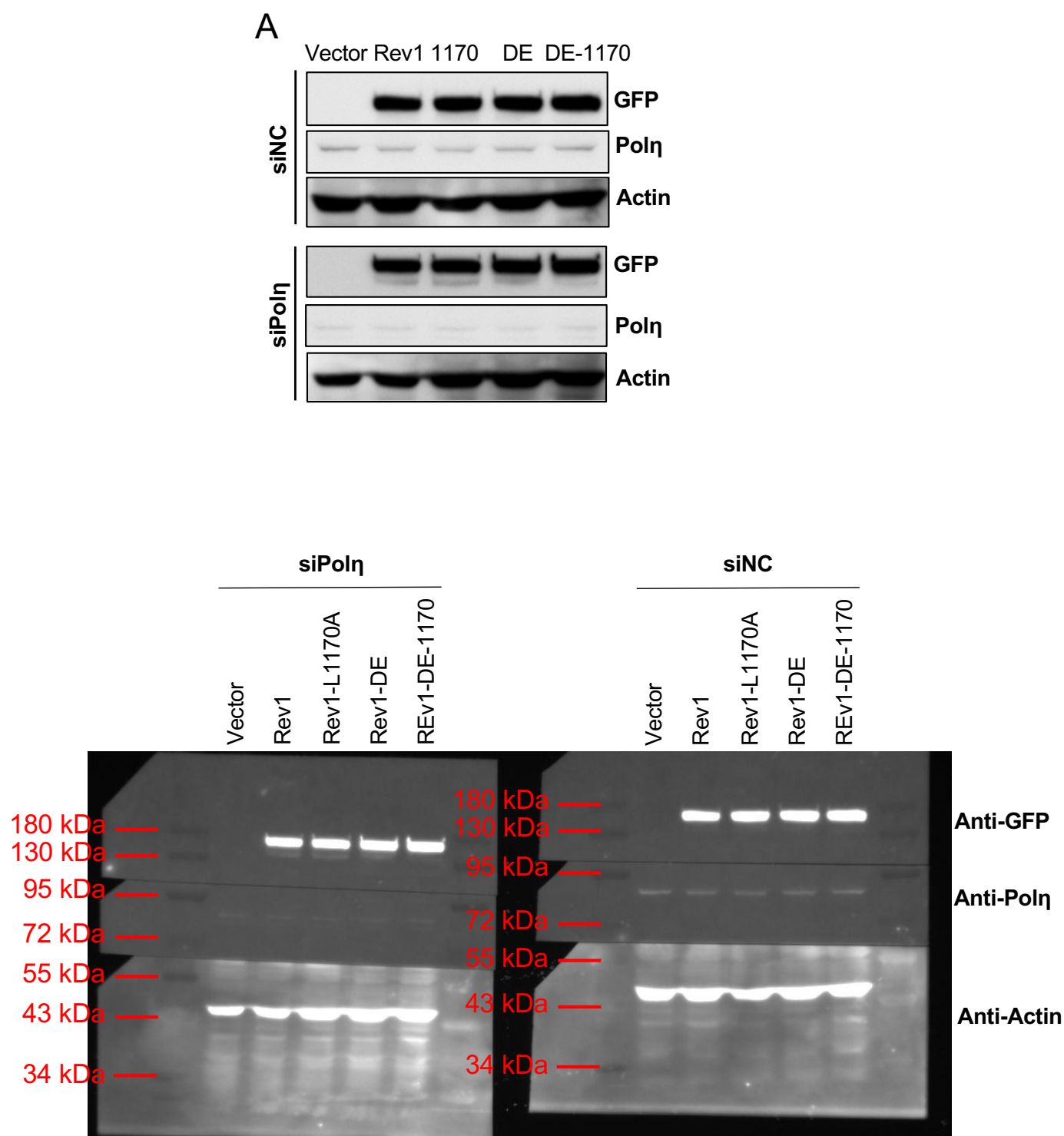

Figure 2A

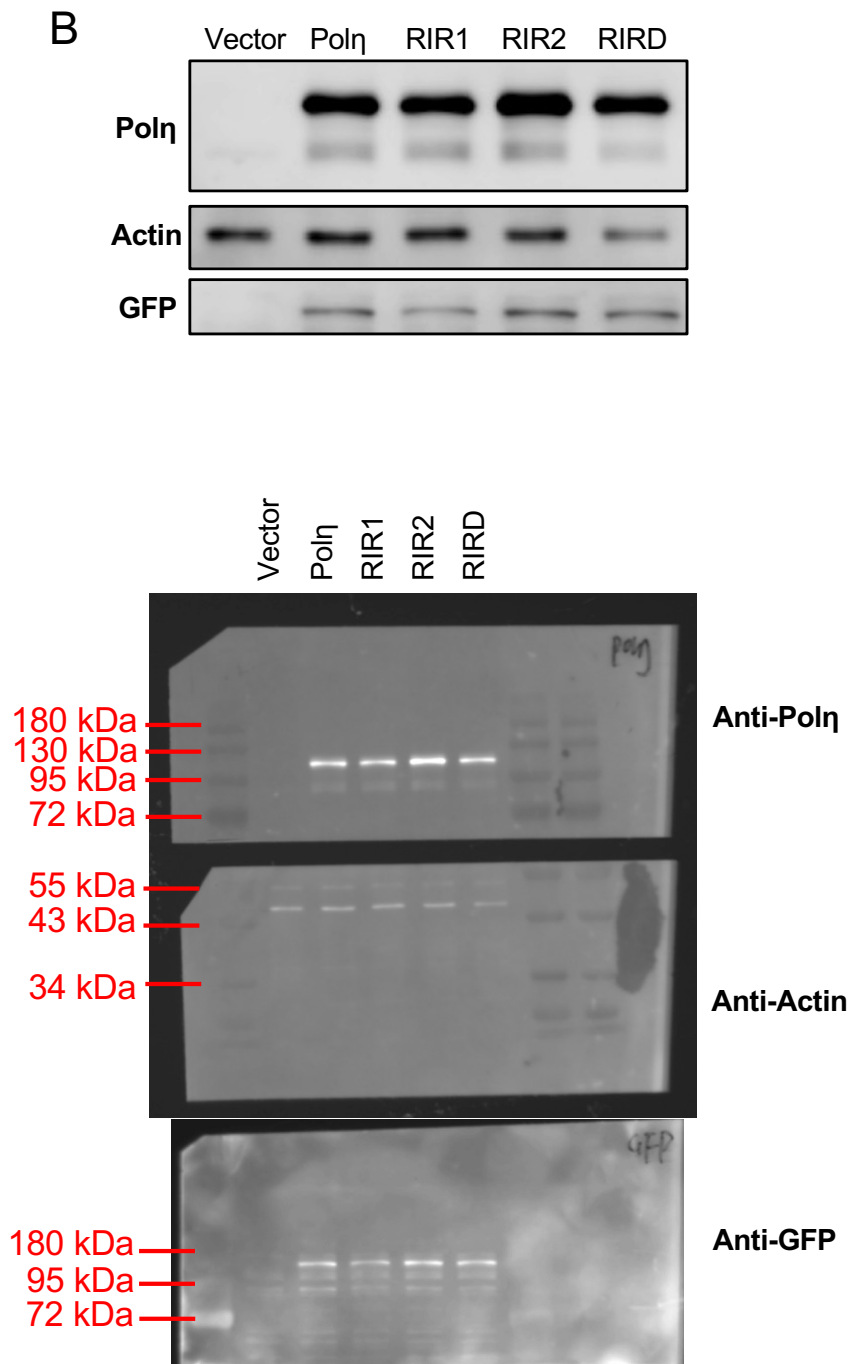

Figure 3B

**B**

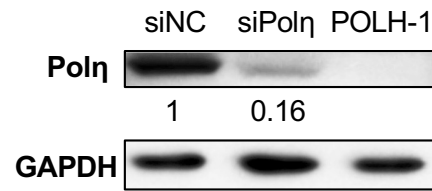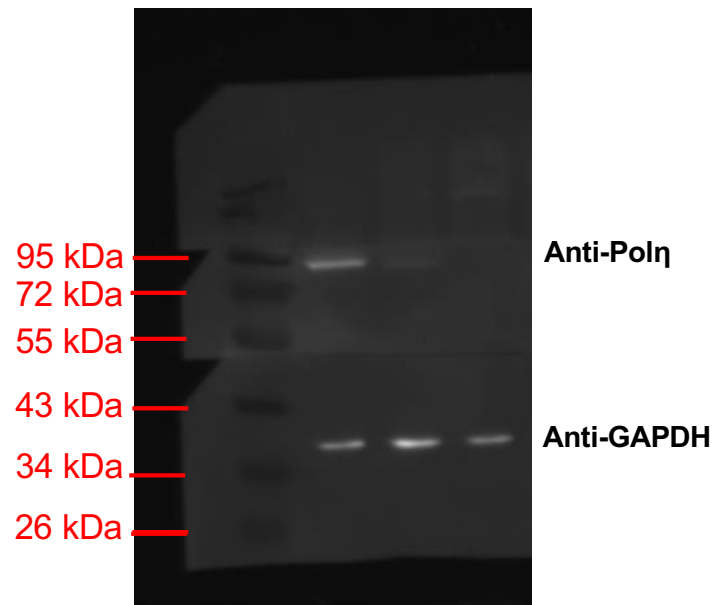

Figure 4B

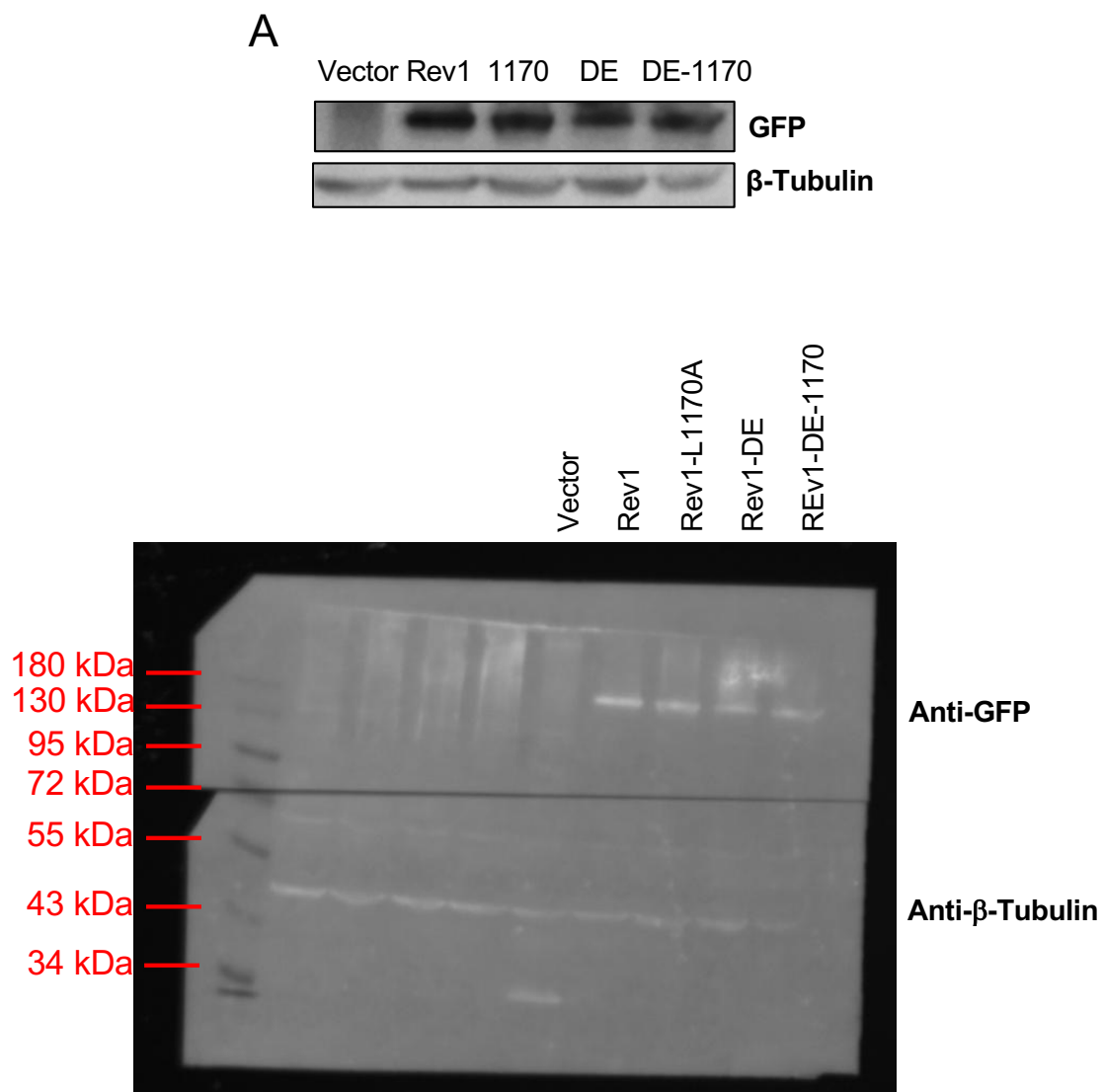

Figure 5A

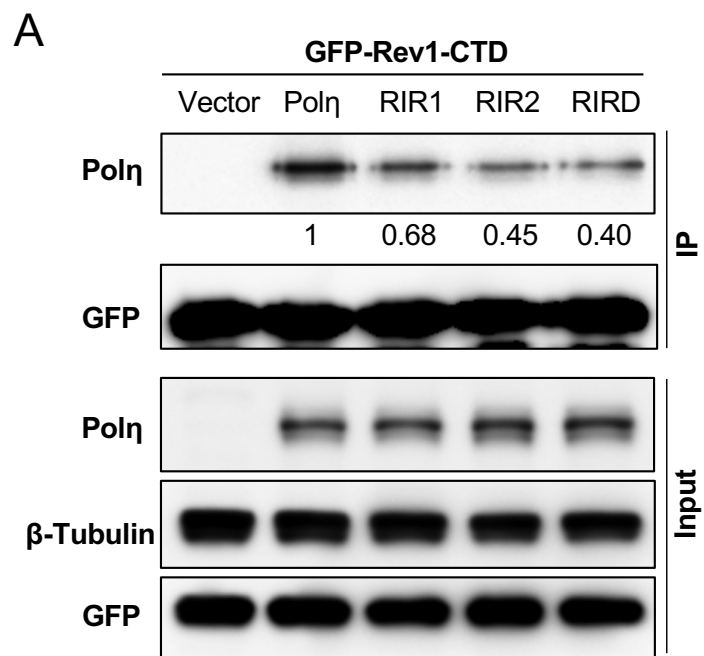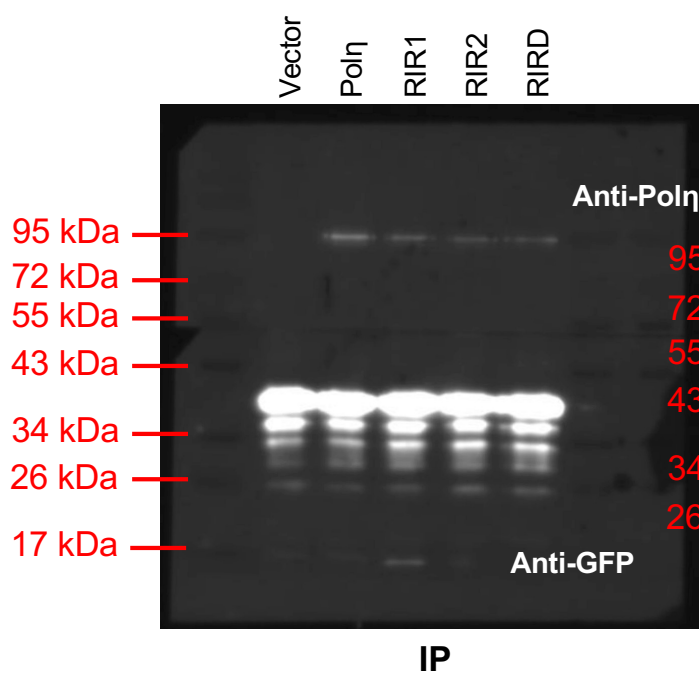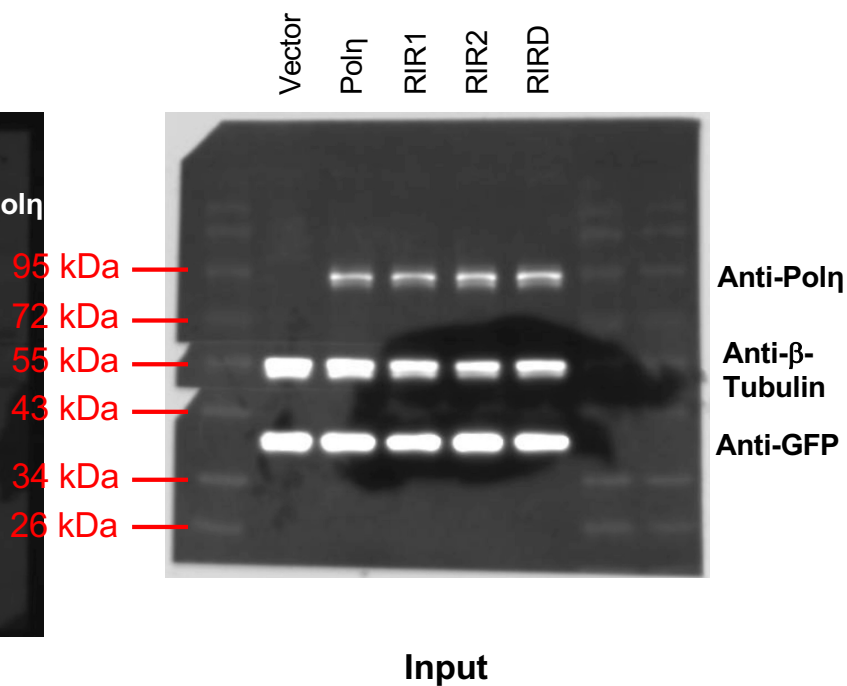

Figure 6A

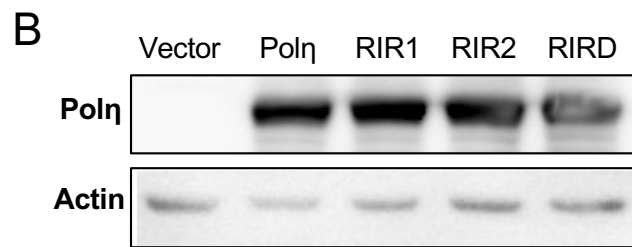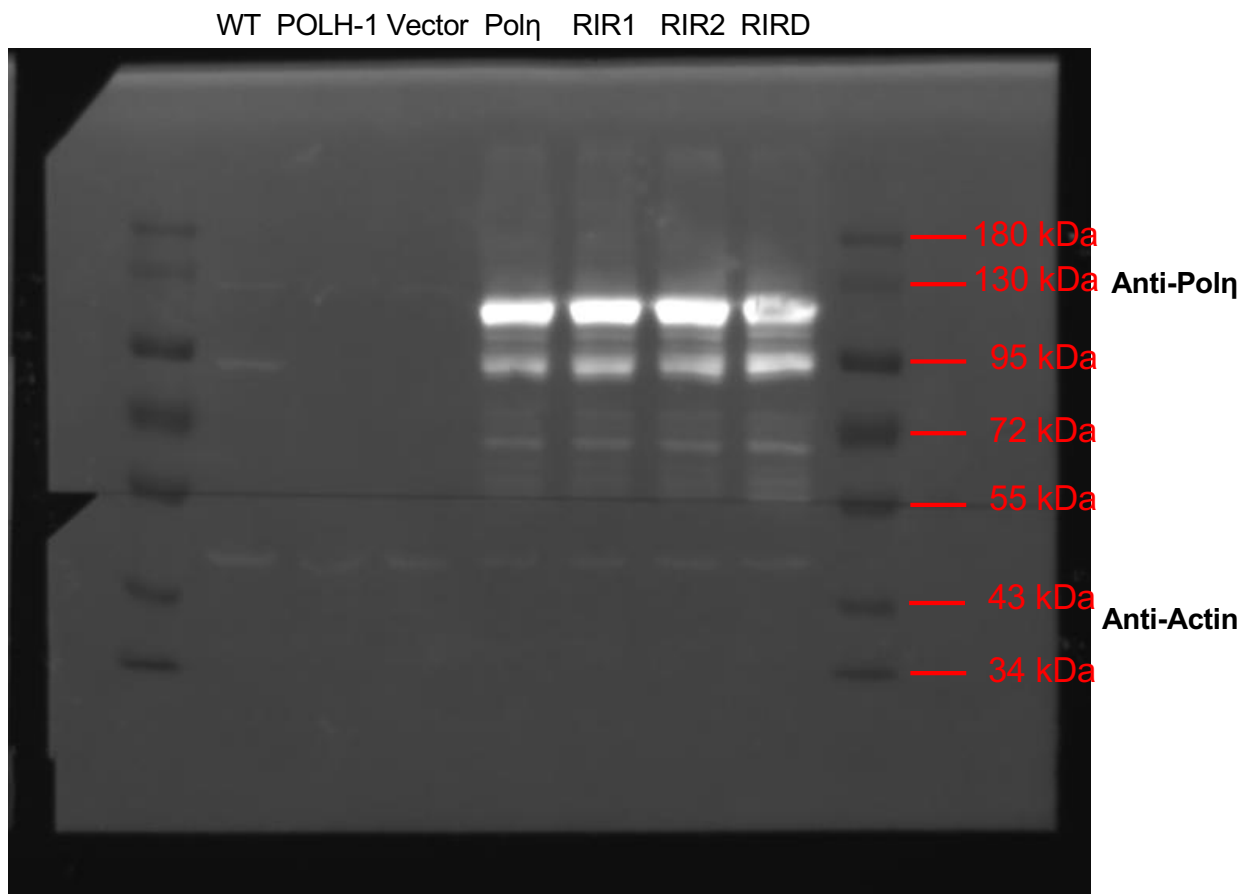

Figure 6B

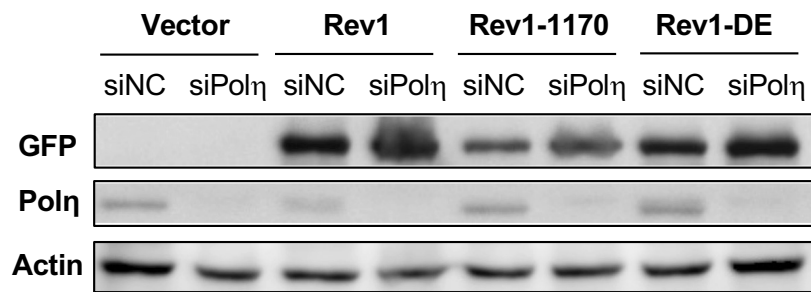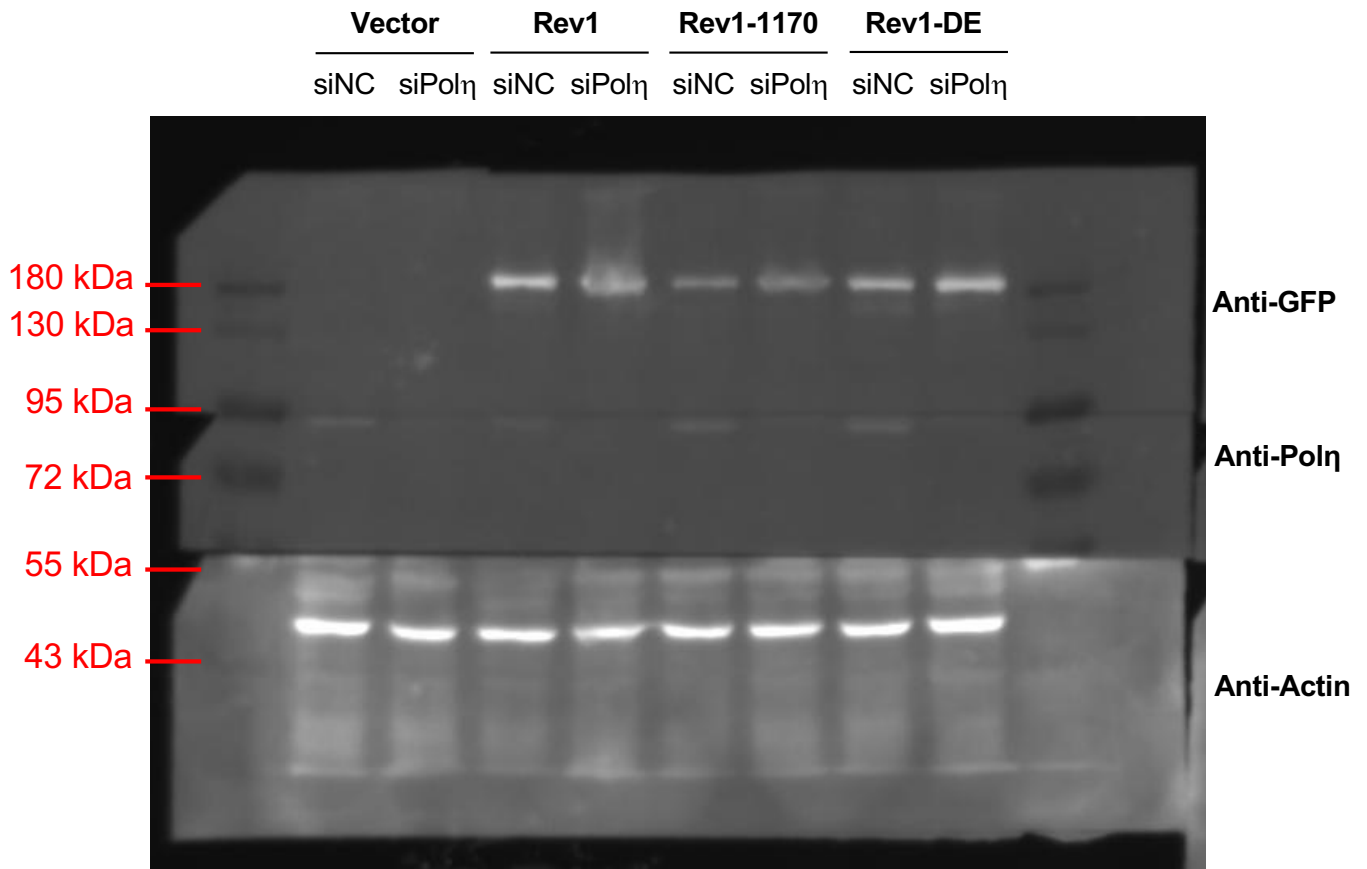

Figure S3

Supplement: Supplementary file 2 — Supplementary Information 2. [file 41598_2021_878_MOESM2_ESM.pdf]
